# Cryo-EM structure of a microtubule-bound parasite kinesin motor and implications for its mechanism and inhibition

**DOI:** 10.1016/j.jbc.2021.101063

**Published:** 2021-08-08

**Authors:** Alexander D. Cook, Anthony J. Roberts, Joseph Atherton, Rita Tewari, Maya Topf, Carolyn A. Moores

**Affiliations:** 1 Institute of Structural and Molecular Biology, Department of Biological Sciences, Birkbeck, University of London, London, United Kingdom; 2 School of Life Sciences, University of Nottingham, Nottingham, United Kingdom

**Keywords:** ATPase, anti-parasitic, cell division, cryo-EM, kinesin, malaria, mechanochemistry, microtubule, motor, *Plasmodium falciparum*, CNB, cover neck bundle, cryo-EM, cryo-electron microscopy, *Hs*K5, *H. sapiens* kinesin-5, MT, microtubule, NBS, nucleotide-binding site, *Pf*K5, *P. falciparum* kinesin-5

## Abstract

*Plasmodium* parasites cause malaria and are responsible annually for hundreds of thousands of deaths. Kinesins are a superfamily of microtubule-dependent ATPases that play important roles in the parasite replicative machinery, which is a potential target for antiparasite drugs. Kinesin-5, a molecular motor that cross-links microtubules, is an established antimitotic target in other disease contexts, but its mechanism in *Plasmodium falciparum* is unclear. Here, we characterized *P. falciparum* kinesin-5 (*Pf*K5) using cryo-EM to determine the motor's nucleotide-dependent microtubule-bound structure and introduced 3D classification of individual motors into our microtubule image processing pipeline to maximize our structural insights. Despite sequence divergence in *Pf*K5, the motor exhibits classical kinesin mechanochemistry, including ATP-induced subdomain rearrangement and cover neck bundle formation, consistent with its plus-ended directed motility. We also observed that an insertion in loop5 of the *Pf*K5 motor domain creates a different environment in the well-characterized human kinesin-5 drug-binding site. Our data reveal the possibility for selective inhibition of *Pf*K5 and can be used to inform future exploration of *Plasmodium* kinesins as antiparasite targets.

Malaria is a massive disease burden worldwide, with an estimated 219 million cases in 2017, a year that also saw the first increase in cases for nearly two decades (https://www.who.int/publications/i/item/9789241565653). With resistance to current frontline therapeutics rapidly rising (https://apps.who.int/iris/handle/10665/274362) (1, 2), new drug targets are urgently needed. Malaria is caused by *Plasmodium* parasites, which are unicellular eukaryotes belonging to the Apicomplexa phylum. Malaria parasites have a complex life cycle involving distinct stages that are transmitted between, and reproduce in, human and mosquito hosts (3). The cytoskeleton plays an important role throughout the parasite life cycle, and the microtubule (MT)-based spindle machinery is involved in the many rounds of mitotic and meiotic replication required for parasite proliferation. Antimitotics are well established as drugs in a variety of settings, notably human cancer (4)—thus, components of the malaria replicative machinery are attractive antiparasite targets. However, given the obligate intracellular nature of malaria parasites, any therapeutic target must be sufficiently divergent to be selectively disrupted compared with host homologues.

Members of the kinesin superfamily are such potential targets. Kinesins are motor proteins that bind to MTs and convert the energy of ATP binding and hydrolysis into MT-based mechanical work. Different kinesin families have specialized functions, such as translocation of cargo along MTs, regulation of MT polymer dynamics, and organization of higher-order MT structures such as mitotic and meiotic spindles (5, 6). MTs are built from heterodimers of the highly conserved α- and β-tubulin, whereas there is approximately 95% sequence conservation of tubulins between *Plasmodium* sp. and *Homo sapiens*, sequence conservation within kinesin families is much lower, typically 40–50%. This raises two important questions: do distantly related members of kinesin families diverge in their molecular properties, and could such sequence divergence allow selective inhibitors to be developed?

The kinesin-5 family is involved in cell division in many organisms, has long been investigated as an antimitotic therapeutic target for human cancer (7), and has also been considered as a target for antifungals (8). Kinesin-5 family members are found in most eukaryotes, including *Plasmodium* sp., and the family is predicted to have been established in the last eukaryotic common ancestor (9). Kinesin-5s form a tetrameric bipolar structure, with two opposing pairs of motor domains that can organize MT arrays such as those found in spindles (10, 11, 12). Several classes of selective *H. sapiens* kinesin-5 (*Hs*K5) inhibitors have been characterized that block motor ATPase activity and bind to allosteric sites in the motor domain. The best studied of these allosteric binding pockets is defined by kinesin-5-specific sequences in a key structural region of the motor domain, loop5 (13). Loop5 is critical for the correct operation of human kinesin-5, since its deletion or mutation disrupts the motor's mechanochemical cycle (14, 15, 16). Furthermore, *Drosophila* kinesin-5 is resistant to the *Hs*K5 inhibitor STLC, but can be sensitized by replacement of loop5 with the cognate *Hs*K5 sequence (17). The loop5-defined allosteric site thus has proven promise in mediating selective inhibition of kinesin-5 family members from different species.

To investigate the idea that kinesin-5 from *Plasmodium falciparum* (*Pf*K5)—the deadliest form of human malaria—could be a selective antimalarial therapeutic target, we characterized the biochemical properties and MT-bound structure of the *Pf*K5 motor domain. We show that the *Pf*K5 motor domain is an ATPase with MT plus-end directed motility, as demonstrated in MT gliding experiments. MT-bound structures of the *Pf*K5 motor domain were determined using cryo-electron microscopy (cryo-EM), and processed in our RELION-based MT image processing pipeline (18). These structures reveal classical kinesin mechanochemistry despite the significant sequence divergence of this motor. This includes ATP-induced subdomain rearrangements that support neck-linker docking to the motor domain and cover neck bundle (CNB) formation, which together are consistent with *Pf*K5 motor domain plus-ended directed motility. Finally, we show that a large insertion in loop5 of the *Pf*K5 motor domain creates a different environment in the well characterized loop5 drug-binding site, revealing the possibility for selective inhibition of *Pf*K5.

## Results

### *Pf*K5ΔL6-MD is a slow ATPase

**Figure 1:**
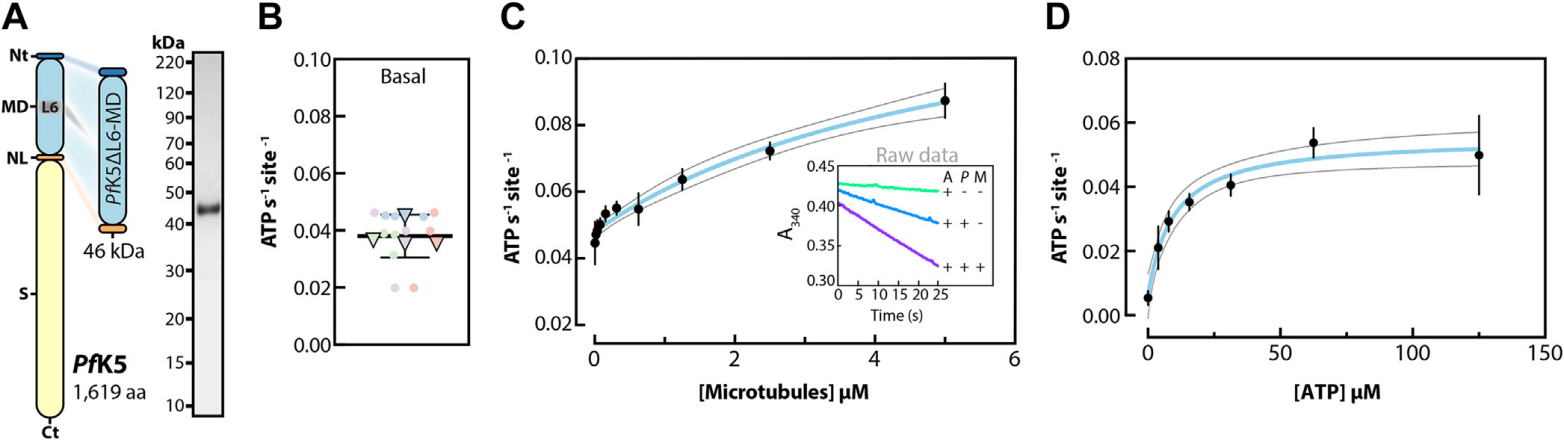
***Pf*K5ΔL6-MD is a slow MT-stimulated ATPase.***A*, *left*, domains of full-length *Pf*K5 and *Pf*K5ΔL6-MD, displaying the N-terminus (Nt), motor domain (MD), neck linker (NL), stalk (S), C-terminus (Ct) aa = amino acids; *right*, Coomassie-stained SDS-PAGE of *Pf*K5ΔL6-MD after purification. *B*, *Pf*K5ΔL6-MD ATPase rate in the absence of MTs. Technical replicates = 12 (*circles*), experimental replicates = 4 (*triangles*), biological replicates (*i.e.*, number of different protein purifications used in the experiments) = 2. The mean of experimental replicates (0.039 ATP s^−1^) and 95% confidence interval are plotted. *C*, *Pf*K5ΔL6-MD MT stimulated ATPase activity. The mean and standard deviation of three experimental replicates (no technical replicates) are plotted. Biological replicates = 2. The fit is plotted as a *blue line*, with corresponding 95% confidence interval plotted as *black lines*. *Inset* displays an example of raw data (A = ATP, *P* = *Pf*K5ΔL6-MD, and M = MTs). *D*, *Pf*K5ΔL6-MD MT stimulated ATPase activity as in (*C*), except with ATP as the substrate variable, and using a constant of 1 μM MT.

To characterize *Pf*K5 mechanochemistry, we first wanted to measure its MT-stimulated ATPase activity. The *Pf*K5 motor domain (*Pf*K5MD, amino acids 1–493) contains a 105 amino acid asparagine and lysine-rich insertion in loop6 that is characteristic of malaria proteins (19) (Fig. 1*A*, left), but which is poorly conserved (16–30% sequence identity) among *Plasmodium* kinesin-5s. We engineered loop6 out of our construct, an approach previously taken by another group (20); this prevented the otherwise near-total precipitation of the nonengineered protein during purification, thereby making all subsequent experiments feasible. We refer to this construct as *Pf*K5ΔL6-MD, and it was purified to 99% purity (Fig. 1*A*, right).

In the absence of MTs, *Pf*K5ΔL6-MD exhibited a low ATP hydrolysis rate (Fig. 1*B*), but addition of MTs stimulated *Pf*K5ΔL6-MD ATPase activity (Fig. 1*C*). Changes in pH or ionic strength showed no or minimal impact respectively on *Pf*K5ΔL6-MD ATPase rate (Fig. S1, *A* and *B*). From these data, the motor k_cat_ and k_m_ of MTs (k_MT_) for *Pf*K5ΔL6-MD were calculated. The k_m_ of ATP (K_ATP_) was also determined (Fig. 1*D*). *Pf*K5ΔL6-MD has a MT-stimulated ATP hydrolysis rate of 0.13 ATP s^−1^, which is slow compared with kinesin-5 from *Sus pombe* (21), *Sus cerevisiae* (22), and *H. sapiens* (23) (with rates of 1.2, 0.5, and 2.9 ATP s^−1^ respectively). However, *Pf*K5ΔL6-MD has similar k_MT_ (5.4 μM) and K_ATP_ (9.5 μM) values compared with previously characterized kinesin-5s (22, 23). Thus, despite substantial sequence divergence, and although it has the lowest ATPase rate observed to date for the family, *Pf*K5ΔL6-MD exhibits overall similar ATPase properties compared with other kinesin-5s.

### *Pf*K5ΔL6-MD generates slow MT gliding

**Figure 2:**
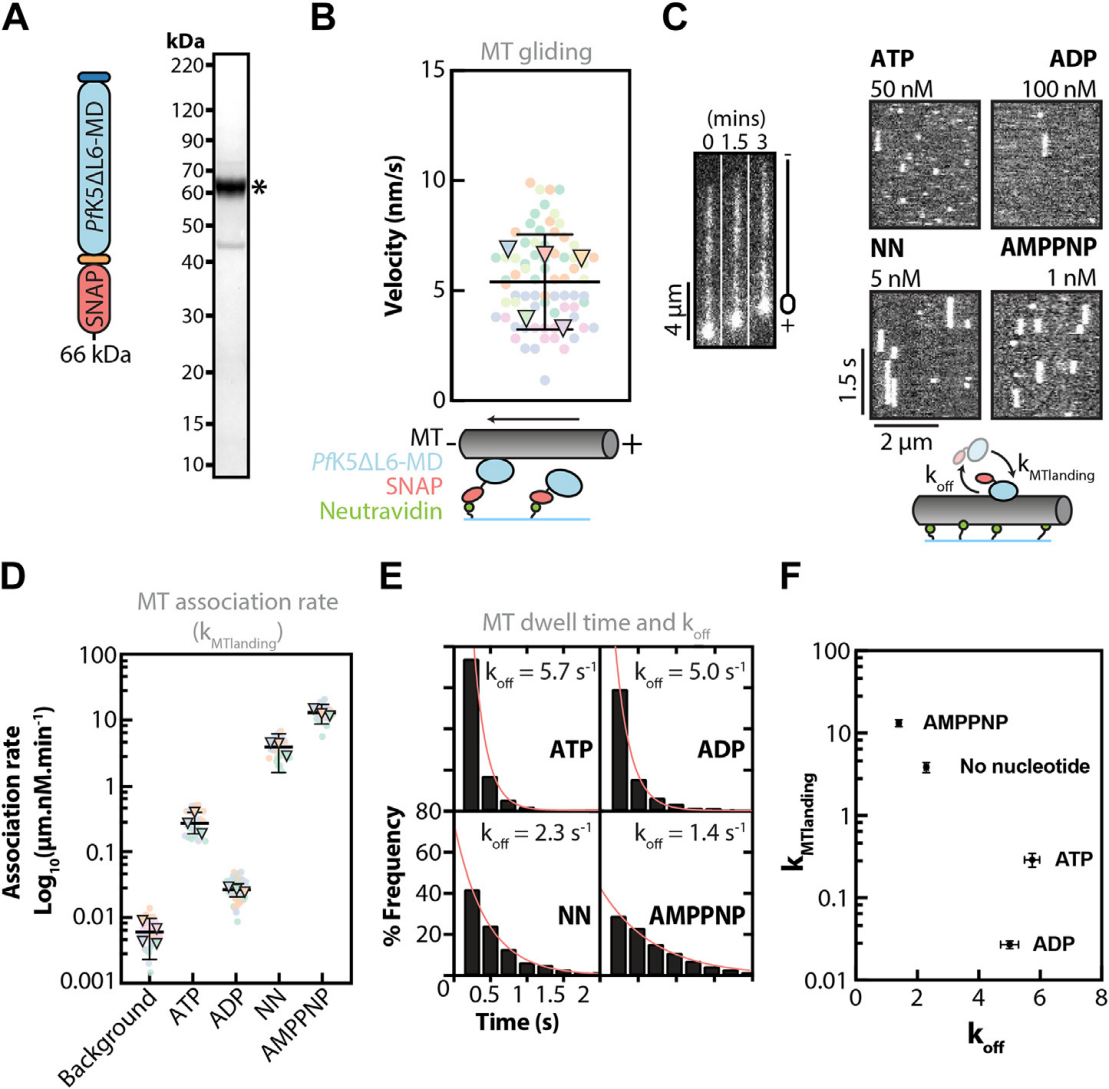
***Pf*K5ΔL6-MD drives plus-end directed MT gliding and its MT interactions are nucleotide modulated.***A*, schematic of the *Pf*K5ΔL6-MD-SNAP fusion protein used for TIRFM experiments, and SDS-PAGE of *Pf*K5ΔL6-MD-SNAP after purification. ∗ indicates the band for *Pf*K5ΔL6-MD-SNAP. *B*, *Pf*K5ΔL6-MD-SNAP driven MT gliding velocity. Technical replicates = 75 (*circles*), experimental replicates = 5 (*triangles*), biological replicates = 3. The mean of experimental replicates (5.4 nm/s) and 95% confidence interval are plotted. Grayscale images on the right are snapshots of a single bright plus-end labeled MT over time. *C*, example kymographs from *Pf*K5ΔL6-MD-SNAP single molecule MT-binding experiments, with each vertical white streak corresponding to a single *Pf*K5ΔL6-MD-SNAP binding event. Concentrations refer to the amount of *Pf*K5ΔL6-MD-SNAP required to see single molecule binding for each nucleotide. *D*, *Pf*K5ΔL6-MD-SNAP MT association rates in different nucleotide states (NN = no nucleotide). For each nucleotide condition, technical replicates (*circles*) and experimental replicates (*triangles*) are plotted, in addition to the mean and 95% confidence interval of experimental replicates. Number of MTs = 34, 22, 46, 28, 29 for the background, ATP, ADP, no nucleotide, and AMPPNP states, respectively. *E*, frequency distribution of *Pf*K5ΔL6-MD-SNAP MT dwell times, in different nucleotide states. Number of experimental replicates = 3; however, frequency distributions are calculated from pooled experimental data. Number of biological replicates = 2. The fit for one-phase exponential decay models is shown, with corresponding decay constant (k_off_). Number of events = 1065, 1084, 1799, 1717 for ATP, ADP, no nucleotide, and AMPPNP states, respectively. *F*, mean MT association (k_MTlanding_) as a function of MT dissociation (k_off_), plotted with 95% confidence intervals.

To determine the motile properties of *Pf*K5ΔL6-MD, we used an MT gliding assay. *Pf*K5ΔL6-MD was expressed with a C-terminal SNAP-tag (*Pf*K5ΔL6-MD-SNAP), purified to 92% purity (Fig. 2*A*), covalently labeled with biotin, and attached to a neutravidin-coated surface. The velocity of fluorescently labeled MTs driven by *Pf*K5ΔL6-MD-SNAP activity was measured. *Pf*K5ΔL6-MD-driven MTs moved at an average velocity of 5.4 nm/s (95% confidence interval = 5–5.9) (Figs. 2*B* and S1*C*). This is slow compared with 23–92 nm/s reported for *Hs*K5-MD (21, 24, 25). However, this slow MT gliding corresponds with the slow rate of ATP hydrolysis observed in the ATPase assay. Inclusion of polarity-marked MTs in the assay further showed that *Pf*K5ΔL6-MD is a plus-end directed motor (Fig. 2*B*).

### *Pf*K5ΔL6-MD single-molecule interactions with MTs

To provide some context for the slow ATPase and MT gliding activity we observed for *Pf*K5ΔL6-MD, we analyzed the interactions of single molecules of fluorescently labeled *Pf*K5ΔL6-MD-SNAP with MTs. We made single-molecule measurements in different nucleotide conditions, to investigate how MT affinity changes with nucleotide state (Figs. 2*C* and S1*D*), using the nonhydrolyzable ATP analogue AMPPNP to mimic the ATP bound state. From these data, we calculated the MT association rate, or k_MTlanding_ (Fig. 2*D*), and MT dissociation rate, or k_off_ of *Pf*K5ΔL6-MD (Fig. 2*E*). This demonstrated that in saturating ADP conditions, *Pf*K5ΔL6-MD-SNAP had a comparatively low k_MTlanding_ and a high k_off_, indicating that *Pf*K5ΔL6-MD-SNAP has low MT affinity when bound to ADP (Fig. 2*F*). In the absence of nucleotide or in saturating AMPPNP conditions, *Pf*K5ΔL6-MD-SNAP had a comparatively high k_MTlanding_ and a low k_off_, showing that when no nucleotide is present, or in its ATP-bound state, *Pf*K5ΔL6-MD-SNAP has high MT affinity.

In saturating ATP conditions, k_MTlanding_ was higher compared with the ADP state; however, k_off_ was similar, suggesting that *Pf*K5ΔL6-MD-SNAP also has relatively low MT affinity in the presence of ATP. Overall, we observe that *Pf*K5ΔL6-MD-SNAP has high MT affinity in the no nucleotide and AMPPNP states compared with ADP and ATP saturating conditions, which is comparable to *Hs*K5 (26). The identification of two high-affinity states provided an opportunity to study the structure of the motor.

### MT-bound *Pf*K5ΔL6-MD structure determination using cryo-EM

To gain molecular insight into the behavior of *Pf*K5ΔL6-MD, its interaction with MTs, and its sensitivity to nucleotide binding, we visualized MT-bound *Pf*K5ΔL6-MD in different nucleotide states using cryo-EM. We calculated 3D reconstructions of *Pf*K5ΔL6-MD bound to MTs in the absence of nucleotide and in an ATP-like state, using AMPPNP (Table 1). To do this we used MiRP (Fig. S2, *A* and *B*), our previously developed pipeline for image processing of MTs with RELION (18, 27). As part of the current study, we have updated MiRP to operate with RELION v3.1 and improved usability of the procedure such that it can be run from the RELION GUI, among other updates (see Experimental procedures and https://github.com/moores-lab/MiRPv2).

**Table 1:** Cryo-EM data collection, 3D image processing and model refinement statistics

Data collection parameter	*Pf*K5ΔL6-MD nucleotide state	
	No nucleotide	AMPPNP
Data Collection and 3D Image Processing		
Magnification	160,000	160,000
Voltage (kV)	300	300
Electron dose (e^−^/Å^2^)	58	58
Pixel size (Å)	1.39	1.39
Number of Images	1320	335
Data Collection Strategy	Manual and automated	Manual
Starting number of particles	48,165	37,843
Final particle number (after particle symmetry expansion)	73,684	206,666
Box size (pixels)	432	432
C1 reconstruction resolution (Å)	4.5	4.8
Asymmetric unit refine resolution (Å)	4	4.4
Model Refinement		
Global Cross-correlation		
Homology model	0.88	0.86
Final model	0.89	0.89
MolProbity		
Homology model	3.46	3.46
Final model	1.13	0.98
QMEAN		
Homology model	−2.21	−2.21
Final model	−0.36	0.16

**Figure 3:**
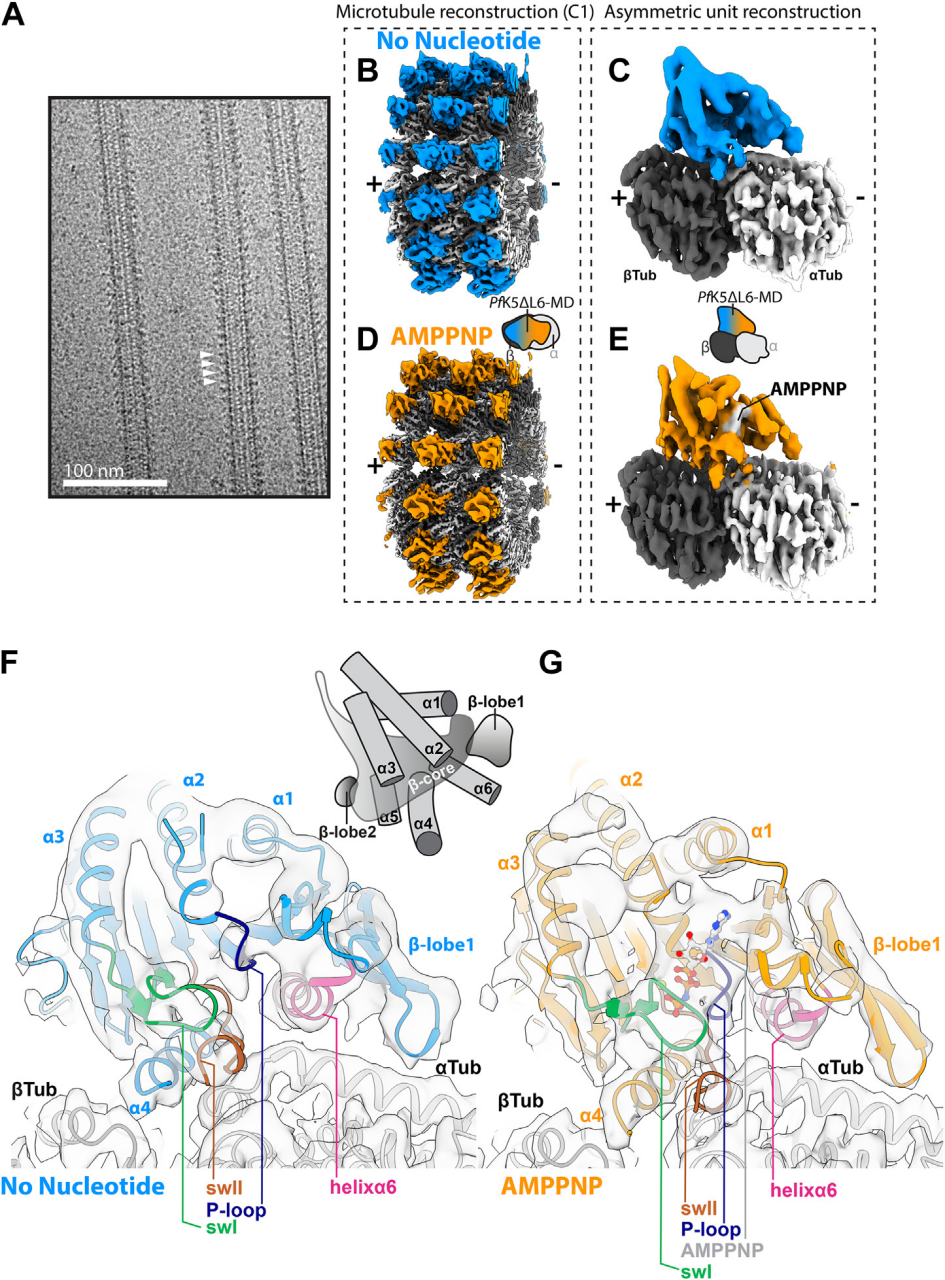
**Cryo-EM 3D reconstruction of *Pf*K5ΔL6-MD MT complexes**. *A*, example micrograph of the *Pf*K5ΔL6-MD bound MTs with 5 mM AMPPNP. *White arrows* indicate *Pf*K5ΔL6-MD decoration present every 8 nm (1 αβ-tubulin dimer). *B–E*, 3D reconstructions have been locally low-pass filtered according to local resolution. *B*, the unsymmetrized (C1) reconstruction of the *Pf*K5ΔL6-MD no nucleotide state bound to MTs, depicting the central portion of the MT reconstruction. *C*, as in (*B*), for the AMPPNP state. *D*, reconstruction of the *Pf*K5ΔL6-MD no nucleotide state bound to αβ-tubulin after asymmetric unit refinement. *E*, as in (*D*), for the AMPPNP state. *F*, Ribbon depiction of the no nucleotide state model in corresponding cryo-EM density. *G*, as in (*F*), for the AMPPNP state. In (*F*) and (*G*), key components of the *Pf*K5ΔL6-MD are labeled and color-coded as indicated.

*Pf*K5ΔL6-MD binds MTs every 8 nm (one *Pf*K5ΔL6-MD per αβ-tubulin) on the ridge of MT protofilaments (Fig. 3*A*), a binding site shared by all other kinesins characterized to date (28, 29). MiRP initially produced reconstructions for the no nucleotide (Figs. 3*B* and S2*C*) and AMPPNP states (Figs. 3*C* and S2*C*) at overall resolutions of 4.8 Å and 4.5 Å, respectively. The resolution in the MT region of both reconstructions is approximately 4.5–5.5 Å—in contrast, the resolution of *Pf*K5ΔL6-MD exhibits a marked falloff as a function of distance from the MT surface (Fig. S2*C*). This is typical of kinesin-MT reconstructions (30, 31) and is attributable to a number of factors, including incomplete occupancy of the MT lattice by the motor that is not immediately apparent in the micrographs, as well as flexibility in the *Pf*K5ΔL6-MD protein itself. We therefore used symmetry expansion and 3D classification focused on *Pf*K5ΔL6-MD from one *Pf*K5ΔL6-MD:αβ-tubulin dimer asymmetric unit to select data with the best *Pf*K5ΔL6-MD occupancy (Fig. S2*A*). We rejected classes that had a clear absence of *Pf*K5ΔL6-MD density, as well as classes that did not contain continuous motor density (Fig. S2*B*). The resulting 3D structures (no nucleotide—Fig. 3*D*, AMPPNP—Fig. 3*E*) were substantially improved, as shown by reduced resolution decay in the motor domain density (Fig. S2, *C* and *D*). The average resolution of these reconstructions was 4.4 and 4 Å for the no nucleotide and AMPPNP states, respectively, with *Pf*K5ΔL6-MD having a resolution range of 4.5–7 Å.

Alignment and superposition of our reconstructions directly reveal nucleotide-dependent differences in these structures (Fig. S3*A*). However, to facilitate interpretation of the differences, we also calculated a *Pf*K5ΔL6-MD homology model and performed flexible fitting of this model within the cryo-EM density, in which individual secondary structure elements were well resolved (Fig. S3*B*). Local cross-correlation scoring showed overall improvement of model fit to density as a result of flexible fitting (Fig. S3*C*) and produced molecular models of each of the *Pf*K5ΔL6-MD:αβ-tubulin dimer complexes (Table 1). These provide a detailed picture of how *Pf*K5ΔL6-MD interacts with both α- and β-tubulin. They also show that this divergent parasite motor has a canonical kinesin fold: it is built from a central β-sheet, sandwiched between three α-helices on each side, and flanked by a small β-sheet (β-lobe1) and a β-hairpin (β-lobe2) (Fig. 3, *F* and *G*). Using these models, we analyzed conformational differences between the no nucleotide and AMPPNP states.

### AMPPNP binding causes *Pf*K5ΔL6-MD nucleotide-binding site closure

The *Pf*K5ΔL6-MD nucleotide-binding site (NBS) is located away from the MT surface, and despite the overall low sequence conservation of *Pf*K5ΔL6-MD compared with *Hs*K5 (Fig. S4), it is composed of three loops containing conserved sequence motifs. These are the P-loop—which interacts with the α- and β-phosphates of bound nucleotide—loop9 and loop11, which contain the switch-I and switch-II motifs, respectively (32).

**Figure 4:**
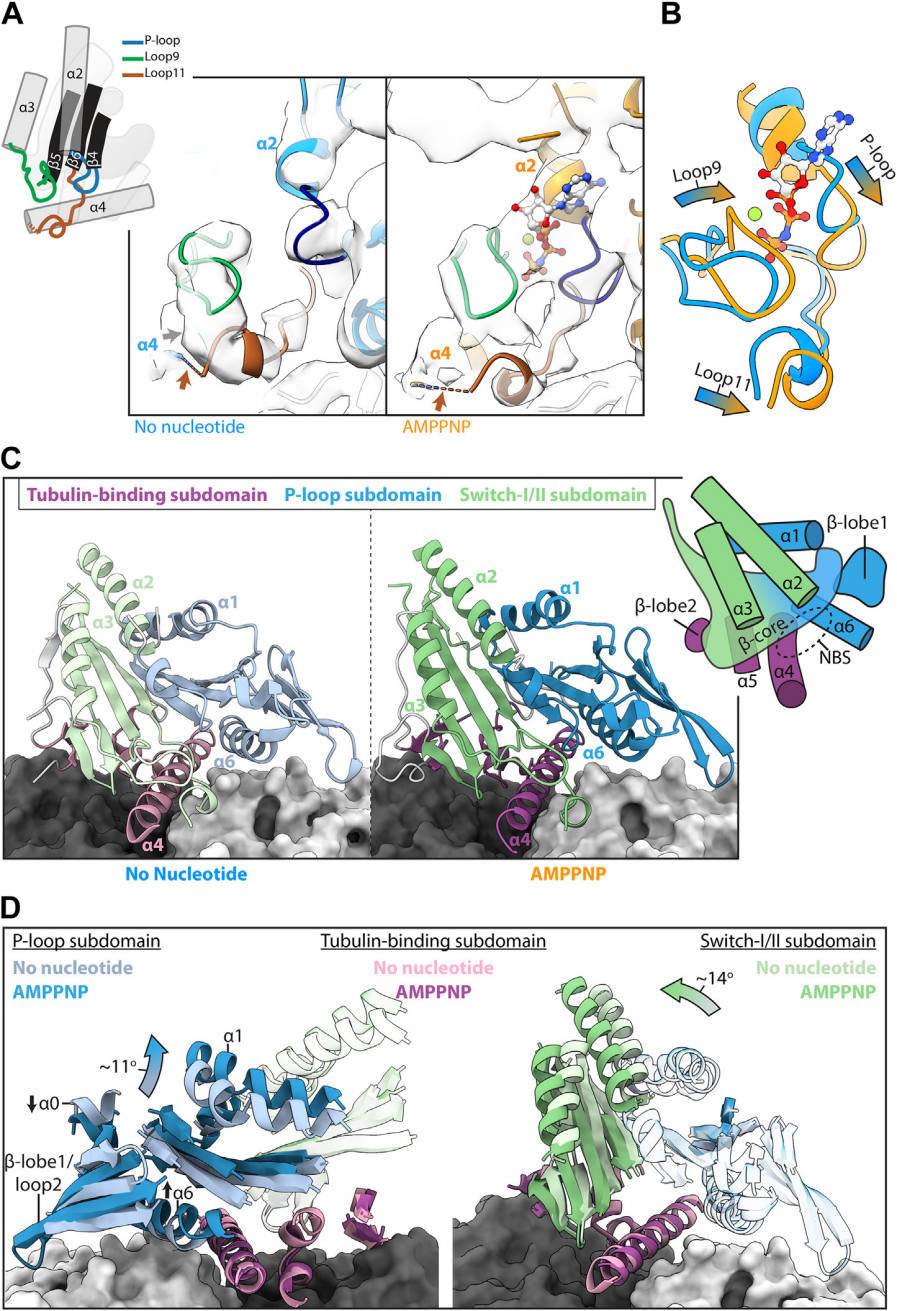
**AMPNPP binding causes *Pf*K5ΔL6-MD subdomain rearrangement and switch loop closure.***A*, rearrangements of *Pf*K5ΔL6-MD switch loops upon AMPPNP binding, with a schematic showing connectivity of the switch loops to motor domain secondary structure elements on the left. Models and corresponding density are displayed on the right, with nucleotide-binding loops colored according to the schematic. The *brown arrow* indicates where there is missing density for loop11, and the *gray arrow* indicates connecting density between loop9 and 11. *B*, comparison of *Pf*K5ΔL6-MD no nucleotide and AMPPNP nucleotide binding loops, demonstrating AMPPNP induced conformational changes. *C*, the *Pf*K5ΔL6-MD no nucleotide state model, coloured according to kinesin subdomain, with α and β-tubulin depicted in light and dark gray surface rendering, respectively. *D*, *Pf*K5ΔL6-MD subdomain rearrangement, showing no overall movement in the tubulin-binding subdomain, with rotations in the P-loop subdomain shown on the *left*, and the switch-I/II subdomain on the *right*.

In the absence of bound nucleotide, density for all three NBS loops is visible in *Pf*K5ΔL6-MD, although density for the P-loop in the no nucleotide state is poorly defined (Fig. 4*A*). In addition, density corresponding to the C-terminal end of loop11, which is approximately 19 Å from the NBS, and contains a two residue *Plasmodium*-conserved insertion, is not visible. In the no nucleotide state, density corresponding to loop9 and the P-loop is well separated, while density is observed connecting loop9 and 11 (Fig. 4*A*). These configurations create an “open” NBS primed for ATP binding formed by a cavity between these three loops. Docking of a model of no nucleotide MT-bound kinesin-1 (33) into *Pf*K5ΔL6-MD density reveals that the kinesin-1 switch loops fit poorly compared with the *Pf*K5ΔL6-MD model, highlighting variations in the “open” NBS conformation between kinesins (Fig. S5*A*). In contrast to the no nucleotide state, in the AMPPNP state, there is clear density corresponding to the bound nucleotide in the *Pf*K5ΔL6-MD NBS (Figs. 4*A* and S5*B*). In addition, the nucleotide is surrounded by loop9 and 11, which have closed around the bound nucleotide to form an NBS that supports ATP hydrolysis (Fig. 4*A*) (34).

Superimposing the *Pf*K5ΔL6-MD no nucleotide and AMPPNP state models by alignment on αβ-tubulin allows visualization of the structural response of the *Pf*K5ΔL6-MD NBS to AMPPNP binding (Fig. 4*B*), while docking of the AMPPNP state model into nucleotide density and vice versa confirms the structural differences between the two states (Fig. S5*C*). Even while the resolution is not sufficient to determine the exact conformation of each of these loops, it is very clear that in the presence of AMPPNP, all three NBS loops move, with loop9 and the P-loop coming closer together, thereby burying the nucleotide. In summary, AMPPNP binding to *Pf*K5ΔL6-MD causes a conformational rearrangement that forms a closed, catalytically competent NBS.

### AMPPNP binding causes *Pf*K5ΔL6-MD subdomain rearrangement

What are the consequences of these NBS rearrangements on the structure of *Pf*K5ΔL6-MD? The structure of *Pf*K5ΔL6-MD can be subdivided into three distinct subdomains (35), which are predicted to move with respect to each other during the motor's MT-based ATPase cycle (Fig. 4*C*). The tubulin-binding subdomain (Fig. 4*C*, purple hues) consists of MT-binding elements in which helixα4 binds a shallow cavity at the intratubulin dimer interface. In addition, helixα5 and β-lobe2 contact β-tubulin. The remaining two subdomains, the P-loop subdomain (Fig. 4*C*, blue hues) and switch-I/II subdomain (Fig. 4*C*, green hues), contain approximately half of the central β-sheet each, along with adjacent secondary structure elements. The NBS is located at the junction of these subdomains (Fig. 4*C*).

We measured the relative rotation of each helix (Table S1) in the transition from no nucleotide to AMPPNP states. This reveals the rearrangement of the P-loop and switch-I/II subdomains around a static MT-binding domain (Fig. 4*D*). The P-loop subdomain pivots such that helixα0 moves toward the MT surface, while helixα6 and the majority of the subdomain move away from the MT surface. The switch-I/II subdomain rotates such that its constituent secondary structure elements move toward β-tubulin.

### AMPPNP binding supports *Pf*K5ΔL6-MD neck linker docking and cover neck bundle formation

**Figure 5:**
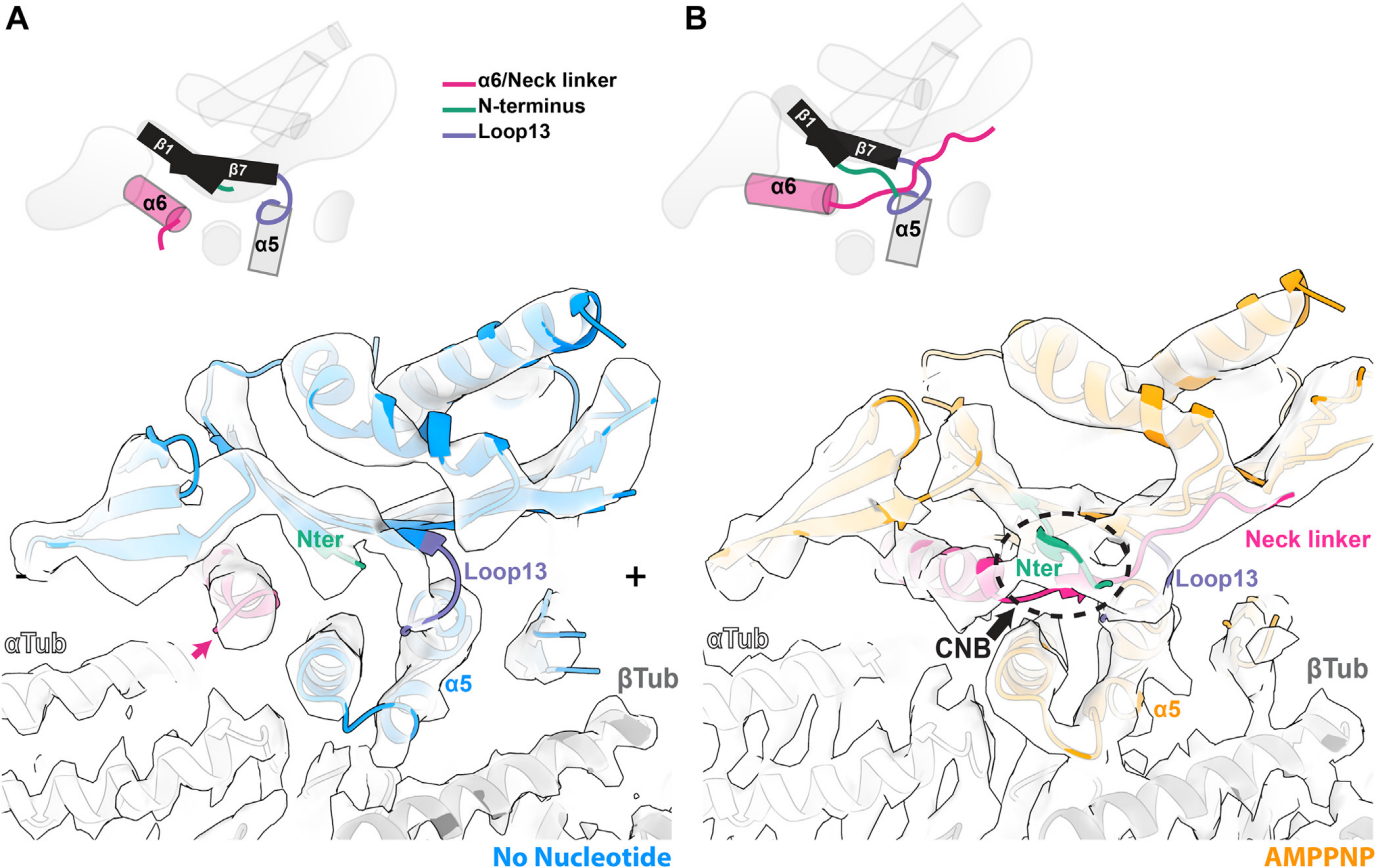
**AMPNPP binding supports *Pf*K5ΔL6-MD cover neck bundle formation.***A*, no nucleotide *Pf*K5ΔL6-MD model and corresponding cryo-EM density of the cover neck bundle region. Helixα6/neck linker, the N-terminus, and loop13 are colored according to the schematic. Note the lack of density for the neck linker at the terminus of helixα6. +/− symbols denote the MT polarity. *Pink arrow* indicates the lack of density for an ordered neck linker. *B*, as in (*A*) for the AMPPNP state (at equivalent density thresholds), showing density corresponding to the neck linker docked along the motor domain and cover neck bundle formation (CNB), which is highlighted by the *dotted black circle*.

What are the consequences for these subdomain rearrangements for the functional output of *Pf*K5ΔL6-MD? Approximately 18 Å away from the MT surface, and 27 Å away from the NBS, is the neck linker. The neck linker is a C-terminal peptide extending from helixα6, which links the motor domain to the kinesin stalk, and which is relatively conserved in the kinesin superfamily (Fig. S6*A*). In the *Pf*K5ΔL6-MD no nucleotide reconstruction, no density is observed extending from helixα6, indicating that the neck linker is disordered in this nucleotide state (Fig. 5*A* and S6*B*). At equivalent thresholds in the AMPPNP state, however, there is clear density corresponding to the neck linker at the C-terminus of helixα6 that extends along the motor domain in the direction of the MT plus-end (Fig. 5*B*). Docking of the AMPPNP state model into no nucleotide density confirms a lack of density to accommodate the neck linker, while density is unaccounted for when the no nucleotide model is docked in AMPPNP density (Fig. S6*C*). In addition, density corresponding to the *Pf*K5ΔL6-MD N-terminus is also visualized in the AMPPNP state; this is consistent with formation of backbone interactions between this region and the neck linker, to form short β-strands known as the CNB, which is characteristic of plus-end directed kinesins (36) (Fig. 5*B*, dotted black oval; S6B). No such CNB density is observed in the no nucleotide reconstruction (Fig. S6*B*). Neck linker docking is enabled by the above-described rotation of the P-loop subdomain, which moves the N-terminus and the central β-sheet away from helixα5/loop13 in the static MT-binding subdomain (Fig. 4*D*) (37, 38, 39, 40). This creates a cavity between loop13 and the N-terminus, known as the docking cleft, which enables neck linker docking. Thus, *Pf*K5ΔL6-MD subdomain rearrangement causes opening of the docking cleft upon AMPPNP binding, a structural transition that is consistent with the ability of *Pf*K5ΔL6-MD to drive ATP-dependent plus-end directed gliding motility (35).

### MT-binding interface

**Figure 6:**
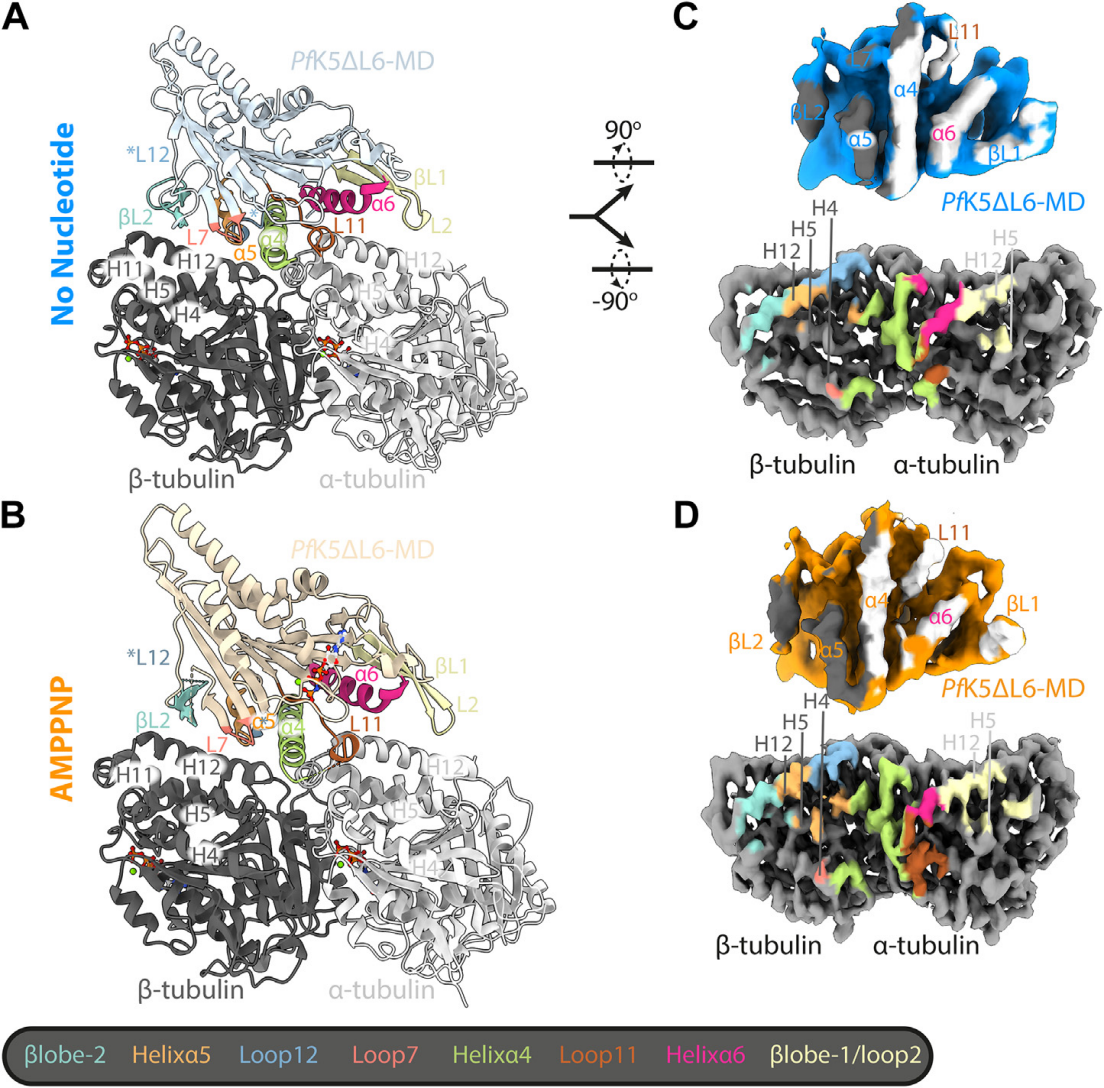
***Pf*K5ΔL6-MD has an altered MT interface.***A*, The *Pf*K5ΔL6-MD no nucleotide state model in ribbon and *coloured light blue* with secondary structure elements partaking in the MT interface colored according to the key below. *B*, as for in (*A*), for the *Pf*K5ΔL6-MD AMMPNP state model, colored in *light orange*. *C*, *upper image*, no nucleotide *Pf*K5ΔL6-MD density, rotated 90° from (*A*), and colored according to αβ-tubulin when a particularly area of density is <7 Å away. *Lower image*, αβ-tubulin density from the no nucleotide state, colored according to different *Pf*K5ΔL6-MD secondary structure elements, as outlined in the key. *D*, as in (*C*), for the *Pf*K5ΔL6-MD AMPPNP state.

*Pf*K5ΔL6-MD binds to one αβ-tubulin dimer, with helixα4 centered at the intradimer interface (Fig. 6, *A* and *B*). At the *Pf*K5ΔL6-MD-binding site, the sequence of the *Sus scrofa* αβ-tubulin used in our reconstruction is identical to that of *P. falciparum* αβ-tubulin (Fig. S7), facilitating a more detailed investigation of this interface. To analyze which *Pf*K5ΔL6-MD secondary structure elements interact with αβ-tubulin in the no nucleotide state, we colored an αβ-tubulin surface representation according to different *Pf*K5ΔL6-MD secondary structure elements; we also colored *Pf*K5ΔL6-MD according to proximity to α or β-tubulin (Fig. 6*C*). This analysis shows that helixα4 interacts with both α- and β-tubulin, while βlobe1, loop11, and helixα6 interact with helices 4, 5, and 12 of α-tubulin, and loop7, βlobe2, and helixα5 interact with similar secondary structure elements in β-tubulin.

Much of this *Pf*K5ΔL6-MD-MT interface is similar between the no nucleotide and AMPPNP states (Fig. 6*D*, Table S2). However, subdomain rearrangement and NBS closure in the AMPPNP state result in some changes. The largest of these occurs at the α-tubulin interface, where rotation of the P-loop subdomain decreases the interaction of helixα6 with α-tubulin, and positions βlobe1 closer to α-tubulin, increasing its interface area. The interface area of loop11 also increases in the AMPPNP state. Interestingly, βlobe1/loop2 forms an interface area with α-tubulin of 181 and 145 Å^2^ in the no nucleotide and AMPPNP states, respectively. Taken together, this shows that the *Pf*K5ΔL6-MD-MT interface is similar to that observed for other kinesin-5s, although the extent to which βlobe1/loop2 interacts with α-tubulin differs between different family members (8, 21, 41).

### *Pf*K5ΔL6-MD loop5 forms a unique putative drug-binding site

**Figure 7:**
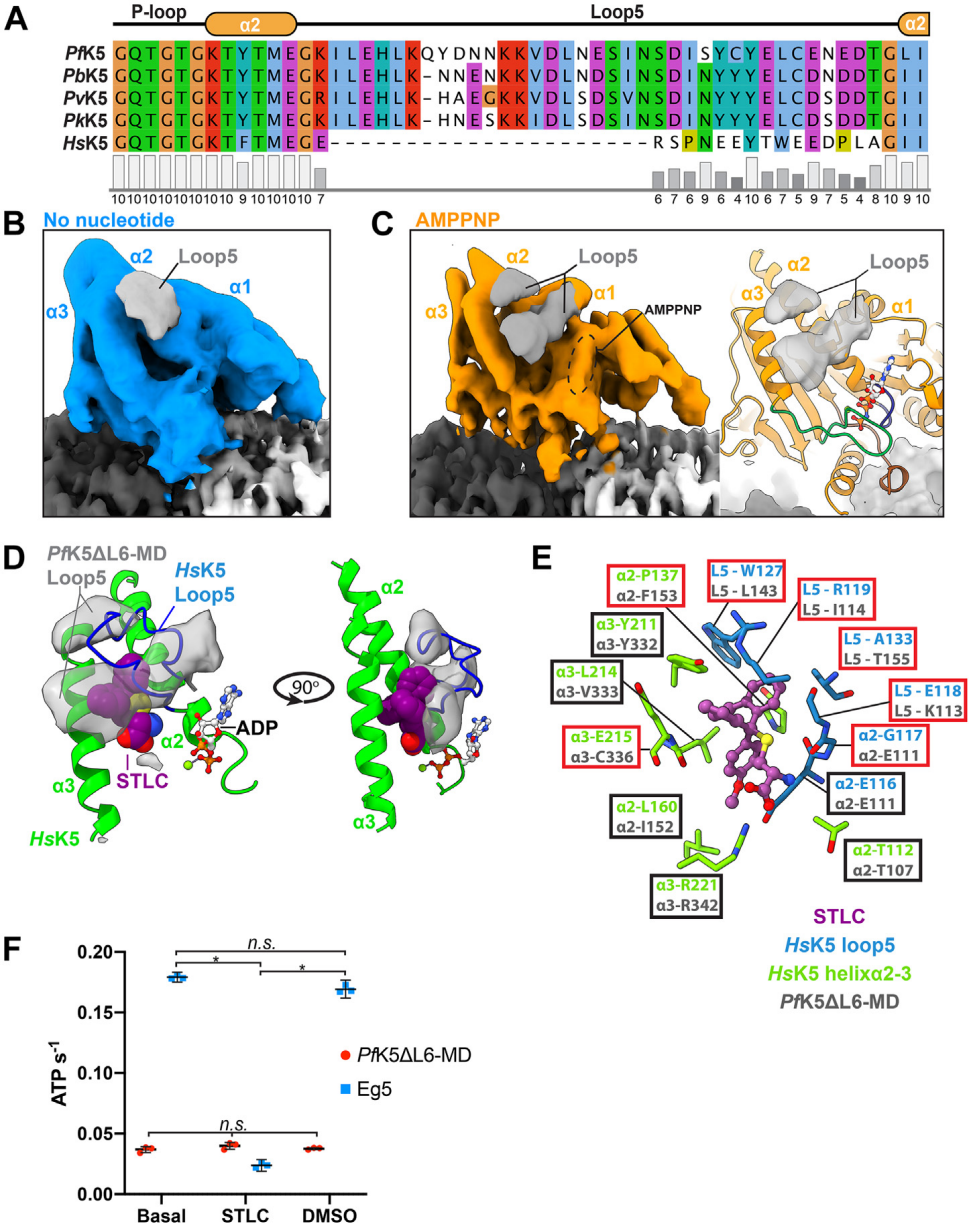
***Pf*K5ΔL6-MD loop5 alters the environment of the kinesin-5 drug-binding site.***A*, primary sequence alignment of loop5 from *Hs*K5 (UniProt ID: P52732), *Pf*K5 (O77382) and various other *Plasmodium* species (*Pv* = *vivax* (A0A564ZV10), *Pk* = *knowlesi* (A0A1Y3DTC2), *Pb* = *berghei* (A0A122I4M3)). Conserved positions are colored according to the ClustalX scheme, and a conservation score as calculated in Jalview is given below. *B*, Loop5 density (*gray*) in the no nucleotide state, compared with other *Pf*K5ΔL6-MD density (*blue*). *C*, Loop5 density (*gray*) in the AMPPNP state, compared with other *Pf*K5ΔL6-MD density (*left*), and the *Pf*K5ΔL6-MD model (*right*). *D*, the STLC bound *Hs*K5 crystal structure in lime (PDB ID: 2WOG (89)), which was rigid body fitted into the *Pf*K5ΔL6-MD AMPPNP state map. STLC is colored *purple*, and *Hs*K5 loop5 is colored *blue*. *Pf*K5ΔL6-MD loop5 cryo-EM density is depicted in *gray*. *E*, conservation of residues partaking in STLC binding between *Pf*K5ΔL6-MD and *Hs*K5, based on primary sequence alignment in (*A*). Using PDB ID: 2WOG, *Hs*K5 residues contacting STLC were found using Chimera, and are displayed, with loop5 residues colored *blue*, and residues from helicesα2-3 colored *green*. Equivalent *Pf*K5 residues are shown in gray with *Hs*K5 residue labels. Nonconserved residues are displayed in *red boxes*, with conserved ones shown in *black boxes*. *F*, ATPase rates of *Pf*K5ΔL6-MD and *Hs*K5 rate in the absence of MTs, with either no treatment, + 20 μM STLC, or DMSO (with the same % v/v as the % v/v of STLC). Statistical relationships were tested using a one-way ANOVA, followed by a post-hoc Tukey's multiple comparison test.

Loop5 plays an important role in the mechanochemistry of *Hs*K5 (14, 16), and forms the drug-binding pocket of *Hs*K5-specific inhibitors (17). It is a solvent exposed loop that creates a break in helixα2 and protrudes from the surface of the motor domain away from the MT. The loop5 sequence is well conserved between *Plasmodium* species (76–91% sequence identity) and is longer compared with *Hs*K5 (Fig. 7*A*). In the no nucleotide state of *Pf*K5ΔL6-MD, some very poorly defined density corresponding to loop5 can be seen at a low threshold (Fig. 7*B*), suggesting that this region is largely disordered in this state.

In the AMPPNP state, however, clear density corresponding to loop5 can be seen, which forms two distinct regions. This density extends at an angle from helixα2, forming elongated density projecting from the motor domain between helixα1 and helixα3 (Fig. 7*C*). This region is at lower resolution than other parts of the reconstruction, possibly owing to intrinsic flexibility, but also possibly because of the residual resolution gradient toward the outer surface of *Pf*K5ΔL6-MD (Fig. S2*C*). There is no secondary structure-like density in this region of the motor, consistent with sequence-based predictions (Fig. S4) and, therefore, a model for *Pf*K5ΔL6-MD loop5 was not calculated.

Strikingly, however, the density corresponding to loop5 in the AMPPNP state does not protrude away from the surface of the motor but appears to cover the site between helicesα2 and 3, equivalent to the well-described inhibitor-binding site in *Hs*K5. Docking of a crystal structure of *Hs*K5 bound to the well-characterized inhibitor STLC in the *Pf*K5ΔL6-MD density reveals the poor match between *Hs*K5 loop5 and the *Pf*K5ΔL6-MD loop5 density (Fig. 7*D*). This also suggests that, although residues outside of loop5 involved in interactions with STLC are largely conserved between *Hs*K5 and *Pf*K5 (Fig. 7*E*), loop5 of *Pf*K5ΔL6-MD might alter the environment of this putative drug-binding site. To test this idea, we measured whether the ATPase activity of *Pf*K5ΔL6-MD was susceptible to inhibition by STLC (42). Consistent with our structural prediction, while STLC inhibits *Hs*K5 ATPase activity, it does not inhibit *Pf*K5ΔL6-MD (Fig. 7*F*). Thus, despite the conserved aspects of *Pf*K5ΔL6-MD mechanochemistry uncovered by our data, evolutionary divergence between *Pf*K5ΔL6-MD and *Hs*K5 mediates differential inhibition of these kinesin-5 motors.

## Discussion

We have determined the biochemical properties and MT-bound cryo-EM structures of a spindle-associated kinesin-5 motor from the malaria parasite. Despite considerable divergence from the human host kinesin-5 sequence, our *P. falciparum* kinesin-5 *Pf*K5ΔL6-MD construct shares with *Hs*K5 a comparatively slow MT-stimulated ATPase, plus-end directed MT gliding activity, and nucleotide-dependent conformational changes that support plus-end directed motility. Significantly, however, our structures revealed a different configuration of the well characterized loop5-defined drug-binding pocket. Further, we also showed that *Pf*K5ΔL6-MD exhibits no sensitivity to the classical *Hs*K5 loop5-binding drug STLC.

The steady-state ATPase activity of *Pf*K5ΔL6-MD is ∼340 times slower than *H. sapiens* kinesin-1 (40), 3–25 times slower than other members of the kinesin-5 family (22, 23, 41), and its MT gliding activity is similarly and proportionally slow. Our use of mammalian brain tubulin rather than native *P. falciparum* tubulin might, in principal, contribute to this—however, αβ-tubulin is well conserved between *S. scrofa* and *P. falciparum*, and the two species have identical residues at the kinesin-binding site (Fig. S7), suggesting that tubulin source is unlikely to influence *Pf*K5ΔL6-MD activity. In further support of this, experiments comparing ATPase rates of a yeast kinesin-5 motor domain interacting with mammalian and yeast tubulin showed no difference (41). A previous study of *P. falciparum* and *vivax* kinesin-5s also observed slow ATPase rates for these motors (20). These findings are reminiscent of the properties of other kinesin-5s and indeed, this may be critical for their function—substitution of the slow motor activity of vertebrate kinesin-5 with faster kinesin-1 was functionally disruptive in the complex context of the spindle (43). This suggests that *Pf*K5—like other kinesin-5s—operates in motor ensembles, where slow-moving teams of *Pf*K5 collaborate to drive MT organization (11).

The malaria kinesin-5 protein we studied was engineered to remove a low-complexity region in loop6, a strategy that had previously been adopted both in characterizing malaria kinesin-5 (20) and other malaria proteins (44). The insertion point of loop6 lies approximately 40 Å from the NBS and, although we cannot exclude that removal of this region influences *Pf*K5ΔL6-MD's behavior, our structures clearly demonstrate that the engineered protein adopts a canonical kinesin fold and undergoes a structural response to AMPPNP binding. Loop6 residues are therefore not required for protein folding and fundamental kinesin mechanochemistry. Low-complexity regions such as *Pf*K5 loop6 are very common in malaria proteins and are often found inserted in otherwise well-conserved three-dimensional folds (19). While the role of such low-complexity regions in immune evasion is logical for extracellular parasite proteins (45), it remains unclear if and how such regions modulate intracellular protein function.

Improvements of our MiRP image analysis procedures allowed us to efficiently handle the incomplete binding of *Pf*K5ΔL6-MD along the MTs in our cryo-EM data (Fig. S2) and to clearly visualize MT-bound *Pf*K5ΔL6-MD at 5–6 Å resolution. Our structures showed that *Pf*K5ΔL6-MD exhibits an open-to-closed conformational change in the NBS, kinesin motor subdomain rearrangements, and CNB formation on ATP analogue binding, typical of a classical plus-end kinesin (33, 40, 46). The lowest resolution region of our *Pf*K5ΔL6-MD is its MT-distal surface, which encompasses the potential drug-binding loop5 region. Because we used GMPCPP-stabilized MTs, we do not think that MT lattice discontinuities—that can occur on paclitaxel-stabilized MTs (31, 47)—cause this resolution loss. Rather, loop5 of *Pf*K5ΔL6-MD, which is 21 residues longer than in *Hs*K5 and composed mainly of hydrophilic residues, appears to be intrinsically flexible and thus its conformation is more challenging to capture structurally. Density for loop5 is only well defined in the AMPPNP state and not the no nucleotide state, suggesting it is conformationally sensitive to bound nucleotide, as also observed with *Hs*K5 loop5 (48). Strikingly, density attributable to loop5 impinges on the pocket corresponding to the well-characterized drug-binding site in *Hs*K5 (49) and provides a possible explanation for the lack of sensitivity of *Pf*K5ΔL6-MD to inhibition by the small-molecule STLC. Given the strong sequence conservation in loop5 between different *Plasmodium* species, this encouraging finding raises the possibility of selective inhibition of parasite motors. Indeed, a small-molecule screen identified a compound able to inhibit *Plasmodium* kinesin-5 ATPase activity, but not that of *Hs*K5 (20).

*Plasmodium berghei* kinesin-5 localizes to mitotic and meiotic spindles in blood and mosquito stages of the parasite life cycle (50), consistent with a conserved role for this motor in the parasite cell division machinery. Although we know very little else about the function of this motor, we infer from our biochemical and structural data that *Plasmodium* kinesin-5 is likely to play an MT-organizing role within parasite spindles. Kinesin-5 is not essential during the blood stages of the *Plasmodium* life cycle (50, 51). However, knockout of *P. berghei* kinesin-5 substantially reduces the number of sporozoites in oocysts and mosquito salivary glands. This highlights the operational diversity of replication at different parasite life cycle stages in general and specifically suggests a key role for kinesin-5 in the multiple rounds of mitosis that occur during sporozoite production in the mosquito host (50).

There is increasing focus on tackling malaria not only during the symptomatic blood stages of the parasite life cycle but also by perturbing *Plasmodium* transmission between vector and host to facilitate malaria control at the population level (52). Intriguingly, despite the reduction of sporozoite numbers in kinesin-5 knockout parasites, the residual sporozoites achieved normal infectivity. Nevertheless, the role of kinesin-5 in this life cycle stage sheds light on parasite transmission vulnerabilities. Particularly given the distinct parasite number threshold that supports onward transmission between vector and host (53), combinations of perturbations that reduce overall sporozoite production could enable transmission control. Moreover, the diverse mechanisms by which small molecules can inhibit *Hs*K5 function have demonstrated that some modes of motor inhibition can be more functionally disruptive than preventing MT binding or than removing motor function completely, for example, by trapping it in a tightly bound MT state (21). In fact, tight MT binding is the proposed mechanism for the antifungal small molecules that target *Candida albicans* kinesin-5, despite that motor being nonessential (54). In the context of these promising findings, our data provide a structural basis for future investigations into parasite-specific kinesin inhibitors.

## Experimental procedures

### Protein expression and purification

The *Pf*K5 motor domain (*Pf*K5MD, residues 1–493) was expressed and purified, but with low yield due to precipitation during purification. Therefore, *Pf*K5MD was engineered such that 105 amino acids of the asparagine/lysine-rich insertion in loop6 (residues 175–269) were removed. The resulting construct, which we refer to as *Pf*K5ΔL6-MD, was cloned in a pET-151D-TOPO vector (Invitrogen) with an N-terminal His_6_-tag and TEV protease cleavage site, and each preparation was expressed in 12 L BL21 Star DE3 *Escherichia coli* cells (Invitrogen) grown in LB media. Cells were grown at 37 °C until they reached an optical density of 0.8–1.0 and were then induced with 0.1 mM IPTG for 3 h at 26 °C. Cells were harvested (6300*g*, 15 min, 4 °C) and stored at –80 °C.

Cells were lysed with three passages through a C3 homogenizer (Avestin) in 100 ml IMAC W buffer (50 mM Tris-HCl pH 8, 400 mM NaCl, 2 mM MgCl_2_, 2 mM DTT, 1 mM ATP, 10 mM imidazole pH 8), which was supplemented with 2× cOmplete EDTA-free protease inhibitor tablets (Roche), 15 μg/ml DNase I (Roche), 0.5 mg/ml Lysozyme, 10% v/v glycerol. The lysate was then clarified by centrifugation (48,000*g*, 45 min, 4 °C). *Pf*K5ΔL6-MD was purified from the clarified lysate at 4 °C using an ÄKTA Pure (GE Healthcare) in 1 day, which reduced loss of protein due to aggregation. First, nickel affinity chromatography was performed where the lysate was loaded onto a 5 ml HisTrap Excel column (GE Healthcare), followed by a wash with 20 column volumes (CV) of IMAC W, then reverse elution with 10 CV of IMAC E buffer (same composition as IMAC W, but with 300 mM imidazole). The eluate from this step was concentrated to 10–15 ml with a Vivaspin concentrator (Sartorius). Concentrated sample was then exchanged into IEX W buffer (same as IMAC W, except containing 80 mM NaCl and no imidazole) using a HiPrep 26/10 desalting column (GE Healthcare). Next, anion exchange chromatography (1 ml HiTrap Q HP) was performed, where the flow-through and wash fractions containing *Pf*K5ΔL6-MD were pooled. The His_6_-tag was then cleaved by incubation with 100 μg/ml TEV protease for 2–4 h at 4 °C. TEV-protease and any remaining contaminants were removed using nickel affinity chromatography (1 ml HisTrap HP), where flow-through and wash fractions containing *Pf*K5ΔL6-MD were pooled. *Pf*K5ΔL6-MD was concentrated to 100–150 μl then, using 0.5 ml Zeba 7K MWCO spin columns, was exchanged into T50K20 buffer (50 mM Tris-HCl pH 8, 20 mM KCl, 2 mM MgCl_2_, 2 mM DTT). This purified *Pf*K5ΔL6-MD was snap frozen in liquid nitrogen and stored at –80 °C. *Pf*K5ΔL6-MD purity was measured using Coomassie-stained SDS-PAGE band intensity measurement in Fiji (55).

### Protein labeling

Gibson assembly (56) was used to clone in-frame a C-terminal SNAP-tag on *Pf*K5ΔL6-MD (*Pf*K5ΔL6-MD-SNAP) for use in total internal reflection microscopy (TIRFM) experiments. Expression and purification of *Pf*K5ΔL6-MD-SNAP were performed as for *Pf*K5ΔL6-MD, except the anion exchange chromatography step was altered as follows: concentrated and desalted sample was added to a 1 ml HiTrap Q HP column, washed with 10 CV IEX W buffer, and eluted with a 20 CV gradient elution to 500 mM NaCl. Eluted fractions containing *Pf*K5ΔL6-MD-SNAP were pooled. *Pf*K5ΔL6-MD-SNAP was biotinylated or fluorescently labeled by overnight incubation at 4 °C with SNAP-Biotin or SNAP-Surface Alex Fluor 647 (New England BioLabs) with at least a 3:1 M excess of these labels to *Pf*K5ΔL6-MD-SNAP. Free SNAP-ligand was removed by two repeats of buffer exchange in T50K20 buffer (0.5 ml Zeba 7K MWCO spin columns).

### MT preparation

Purified and lyophilized unlabeled, X-rhodamine labeled, or biotin labeled *S. scrofa* brain tubulin—except X-rhodamine, which was from *Bos taurus*—(catalogue numbers T240C, TL620M T333P, respectively, all >99% pure, Cytoskeleton Inc) was reconstituted to 10 mg/ml in BRB80 (80 mM PIPES pH 6.8, 2 mM MgCl_2_ 1 mM EGTA pH 6.8), centrifuged at 611,453*g* for 10 min at 4 °C, and the supernatant snap frozen in liquid nitrogen and stored at –80 °C. Double cycle GMPCPP polymerization was performed as follows: reconstituted tubulin was supplemented with 1 mM GMPCPP (Jena Biosciences) and incubated for 5 min on ice. Tubulin was then polymerized at 4–5 mg/ml for 20 min at 37 °C. MTs were then pelleted at 611,453*g* for 10 min at 23 °C, washed twice, then resuspended, both with BRB80. MTs were then depolymerized on ice for 15 min, and a second round of polymerization performed as above. MTs for the ATPase assay were pelleted on a 50% v/v sucrose/BRB80 cushion after the second polymerization, to aid separation of MTs from unpolymerized tubulin.

For labeled MTs, X-rhodamine and unlabeled tubulin were mixed in a 1:9 ratio, or X-rhodamine, biotinylated, and unlabeled tubulin were mixed in a 1:1:8 ratio and polymerized at approximately 4 mg/ml total tubulin with 1 mM GTP at 37 °C for 20 min 40 μM Paclitaxel (Merck) dissolved in DMSO was then added, followed by a further incubation at 37 °C for 15 min, and then incubation at room temperature for at least 1 day before use. To prepare polarity marked MTs (57), long, dimly labeled MTs (1:9 ratio of X-rhodamine to unlabeled tubulin) were polymerized at 2 mg/ml total tubulin for 2 h with 1 mM GMPCPP. To prepare NEM-tubulin, by which minus-end MT growth is blocked, 8 mg/ml unlabeled tubulin was incubated with 1 mM N-ethyl maleimide (Sigma) on ice for 10 min, then with 100 mM ß-mercaptoethanol (Sigma) for 10 min. To polymerize the bright plus-end MT cap, NEM-tubulin was mixed 1:1 with X-rhodamine tubulin and incubated at 37 °C for 15 min. Finally, long, dim MTs were pelleted (15 min, 17,000*g*, room temperature), the pellet resuspended with bright MT caps, incubated 37 °C for 15 min, then 40 μM paclitaxel added.

### Steady-state ATPase activity

An NADH-coupled ATPase assay (58) containing 1.5 mM Phosphoenolpyruvate (Sigma), 9–15 U/ml pyruvate dehydrogenase (Sigma), 13.5–21 U/ml lactate dehydrogenase (Sigma), and 0.25 mM NADH (Roche) was used to measure *Pf*K5ΔL6-MD ATPase rates. Reactions were performed in T50K40 buffer (50 mM Tris-HCl pH 8, 400 mM KCl, 2 mM MgCl_2_, 2 mM DTT) with 100–200 nM *Pf*K5ΔL6-MD. A_340_ readings were taken on a SpectraMax 384 (Molecular Devices) from 100 μl reactions at 26 °C, with automatic path length correction to 1 cm, taking readings every 10 s for 30 min. Background readings containing no *Pf*K5ΔL6-MD were subtracted from all readings. ATP hydrolysis per second per site was derived with the Beer–Lambert equation, and k_cat_ calculated using the Michaelis–Menten equation with the term K_0_ (rate at substrate concentration of 0).

$$ATP\;s^{-1}\;site^{-1}=\;\frac{\left(k_{cat}-K_{0}\right)\;\left[S\right]}{K_{m}+\left[S\right]}+K_{0}$$

### MT gliding and single-molecule TIRFM assays

TIRFM assays with fluorescently labeled protein were performed in a flow-chamber, created by adhering biotin-PEG coverslips (MicroSurfaces Inc) to glass slides with double-sided tape. To prepare flow chambers for the MT gliding assay, the following treatments were made. (1) 0.75% Pluronic acid +5 mg/ml casein was added for 5 min, and the chamber was washed with T50K20 buffer + 20 μM paclitaxel (T50T). (2) 0.5 mg/ml neutravidin was added for 2 min, then the chamber was washed twice with T50T + 1 mg/ml casein (T50TC). (3) 1–2.5 μM *Pf*K5ΔL6-MD-SNAP was added for 2 min, then washed twice with T50TC. (4) Finally, T50TC supplemented with 20 mM glucose, 300 μM glucose oxidase, 60 μg/ml catalase, 71 mM ß-mercaptoethanol, and 0.5 μM X-rhodamine MTs was added. For single-molecule experiments, the same preparations were performed with the following exceptions. (3) 0.5 μM X-rhodamine/biotin MTs was added for 2 min, then washed twice with T50TC. (4) As above, except 1–100 nM *Pf*K5ΔL6-MD-SNAP-alexa647 was added instead of MTs.

Fluorescent molecules were visualized on an Eclipse Ti-E inverted microscope with H-TIRF illuminator, LU-N4 laser unit, and CFI Apo TIRF 1.49 NA oil objective lens 100× (Nikon) (59). Images were recorded on an iXon DU888 Ultra EMCCD camera (Andor) with 60–100 ms exposures. MT velocity and parameters of *Pf*K5ΔL6-MD-SNAP MT binding were calculated from kymographs generated in FIJI (55). For MT binding, only events ≥3 frames were considered. Mean dwell time/k_off_ was calculated by fitting a one-phase exponential decay model to the data. Background k_on_ rates were measured by randomly sampling areas without MTs in the ADP and ATP states, because these states had high background binding, owing to the high concentrations needed to observe *Pf*K5ΔL6-MD-SNAP-binding events.

### Cryo-EM sample preparation, imaging, and data processing

Nucleotide-free *Pf*K5ΔL6-MD was prepared by incubation with 10 U/ml apyrase (Sigma) for at least 15 min at 4 °C, to remove any nucleotides still present following purification. UltrAuFoil 1.2/1.3 gold (Quantifoil) electron microscopy grids were glow discharged in air for 1 min at 0.3 mpa using a PELCO easiGlow (Ted Pella, Inc.). Using a Vitrobot Mark IV (ThermoFisher), with the chamber set to 23 °C and 95% humidity, 4 μl of 3.5 μM GMPCPP MTs was added to grids and incubated for 30 s, then blotted. Four microliter of 50 μM no nucleotide *Pf*K5ΔL6-MD was then immediately added, incubated for 30 s, then blotted. A second round of *Pf*K5ΔL6-MD was added before blotting and plunge freezing into an ethane slush. AMPPNP *Pf*K5ΔL6-MD was prepared by incubation with 5 mM AMPPNP (R&D Systems) for at least 5 min at 4 °C. Vitrification was performed as above, except using C-flat 2/2 carbon (Protochips) holey grids with 5 μM GMPCPP MTs.

Imaging was manually or automatically performed on a G2 Polara (FEI/ThermoFisher) operating at 300 kV using SerialEM (60). Images were collected on a K2 summit detector in counting mode, with a GIF Quantum LS Imaging Filter (Gatan). The sample was exposed with 58 e^−^/Å^2^ for 18 s and 60 frames collected with a pixel size at the sample of 1.39 Å.

All image processing steps were performed with RELION v3.0 and 3.1 (27, 61) except where otherwise noted. Beam-induced motion in micrographs was corrected using RELION's implementation of MotionCor2, and the CTF was determined for each micrograph using Gctf (62). The start/end coordinates of each MT were manually assigned, and MT particles with a box size of 432 pixels were extracted every 82 Å and normalized. Alignment and asymmetric reconstruction of 14 protofilament (PF) MTs (which are the dominant MT type in GMPCPP preparations (63)) was performed using MiRP (18), as follows, with new MiRP updates noted (Fig. S2). RELION parameters used for MiRP PF sorting, initial seam alignment, and seam checking steps are listed in Table S3. Briefly, PF number assignment for each MT was performed with supervised 3D classification. As part of the MiRP update undertaken during this work, after 3D classification, PF number class assignments for each MT were smoothened by calculating the mode of class assignment over a seven-particle window. Where changes in class assignment occurred within a single MT—due to, for example, changes in PF number or major defects within a single MT—MT regions were subsequently treated as distinct MTs. This improved the homogeneity of each MT, increasing confidence in protofilament number assignment and seam location determination. Initial seam alignment was then performed with several iterations of 3D alignment. This was followed, as previously, by Rot angle and X/Y coordinate fitting—however, a local search step was added to improve Rot angle and X/Y shift assignment. Seam checking *via* supervised 3D classification was then performed, and MTs with less than 50% confidence in seam class assignment were removed. C1 reconstructions were obtained with a 3D auto-refine run (using the parameters for X/Y refine in Table S3, with a solvent mask obtained from a 3D reconstruction of seam checking results), followed by per-particle CTF refinement, Bayesian polishing, and beam-tilt estimation, then a second 3D auto-refine with these new corrections. MiRP was also updated to improve useability, by creating three programs for each MiRP step that can be operated from the RELION v3.1 GUI (https://github.com/moores-lab/MiRPv2).

Symmetrized maps were obtained by first performing 2D classification without alignment (200 classes, T = 8) and selecting well-aligned classes with many particles and an estimated resolution better than 6 Å. A 3D auto-refine run was then performed where 14-fold local symmetry was applied, as previously described (64). To address *Pf*K5ΔL6-MD heterogeneity, 3D classification was performed at the level of a *Pf*K5ΔL6-MD:αβ-tubulin asymmetric unit. For this, symmetry expansion was applied to all particles, then 3D classification (4 classes, T = 256) without alignment and a mask around one *Pf*K5ΔL6-MD site opposite the seam was applied. This resulted in one class with clear *Pf*K5ΔL6-MD decoration, which was selected and subjected to a 3D auto-refine procedure. The MT-bound nucleotide-free and AMPPNP-bound *Pf*K5ΔL6-MD reconstructions are deposited with the Electron Microscopy Data Bank, deposition number 12257 and 12258, respectively.

### Sequence analysis, comparative modeling, and flexible fitting

To obtain a kinesin-5 family sequence alignment, the motor domains of all kinesin-5 family members in the Swiss-Prot database were aligned with MAFFT (65). A hidden Markov model of this alignment was created and queried against the UniProt Reference Proteomes database using HMMER (66). Sequences obtained were then compared with a kinesin profile from the Pfam database (67), and those with less than 400 identities were removed. Finally, the sequences were aligned with MAFFT, using the L-INS-i method (File S1). Secondary structure prediction was performed using Quick2D (68), using various prediction algorithms (69, 70, 71, 72, 73). A residue was assigned helical or beta-sheet identity if three or more prediction algorithms agreed.

The above sequence alignment was used in homology modeling of *Pf*K5ΔL6-MD, using the human kinesin-5 AMPPNP bound crystal structure (PDB ID: 3HQD (34)) as a template. For this, 100 homology models were produced with Modeler v9.2 (74), then scored using QMEAN (75) and the top model selected. Restraints used to model the extended helixα2 and for highly conserved positions in the nucleotide binding site are listed in Table S4. Flexible fitting of *Pf*K5ΔL6-MD secondary structure elements into no nucleotide and AMPPNP cryo-EM reconstructions was performed using Flex-EM (76) (cap shift = 0.15) after rigid body docking of the top homology model using the *Fit in Map* tool in Chimera (77). During this procedure, the NBS (AMPPNP, Mg^2+^, switch-I/II loops, and the P-loop) was defined as a rigid body, while loop regions were treated as “all atoms.” Models of the N-terminus, loops 2, 8, 9, 10, 11, and 12, and the neck linker were predicted using Rosetta, firstly using a coarse method (500 models using cyclic coordinate descent with fragment insertion in the centroid modeling step (78)), then the model with highest cross-correlation was selected for a second prediction (500 models using kinematic closure with a fit to density term in the centroid modeling step (79)). A local all-atom fit to density step was then performed using the Rosetta *Relax* procedure including a fit to density term (80). Finally, the interface between *Pf*K5ΔL6-MD and αβ-tubulin was refined with protein–protein docking restrained by cryo-EM density in HADDOCK (81) as described previously (30), using the PDB ID 3JAT (82) as αβ-tubulin atomic model. SMOC scores were calculated using the TEMPy software package (83, 84). The molecular models of MT-bound nucleotide-free and AMPPNP-bound *Pf*K5ΔL6-MD are deposited with the Worldwide Protein Data Bank, deposition number 7NB8 and 7NBA, respectively.

### Visualization and analysis

Plotting was performed with GraphPad Prism 8. Cryo-EM density and model analysis was done in Chimera (85) and ChimeraX (86). Protein sequence analysis was done in Jalview (87). Protein interface areas were calculated with PDBe PISA v1.52 (88).

## Data availability

Updated MiRP scripts and instructions are available on GitHub: https://github.com/moores-lab/MiRPv2. The MT-bound nucleotide-free and AMPPNP-bound *Pf*K5ΔL6-MD reconstructions are deposited with the Electron Microscopy Data Bank, deposition number 12257 and 12258, respectively. The molecular models of MT-bound nucleotide-free and AMPPNP-bound *Pf*K5ΔL6-MD are deposited with the Worldwide Protein Data Bank, deposition number 7NB8 and 7NBA, respectively. The kinesin-5 family motor domain sequence alignment is provided in File S1.

## Supporting information

This article contains supporting information.

## Conflict of interest

The authors declare that they have no conflicts of interest with the contents of this article.

## Supporting information

Figures S1–S7 and Tables S1–S4

File S1 Kinesin-5 motor domain sequence alignment.
