# Supplementary material for: Cryo-EM structure of a microtubule-bound parasite kinesin motor and implications for its mechanism and inhibition
Source: J Biol Chem. 2021 Aug 8;297(5):101063. doi: 10.1016/j.jbc.2021.101063 (PMC8526983; doi:10.1016/j.jbc.2021.101063)
Supplement: Figures S1–S7 and Tables S1–S4 [file mmc1.pdf]

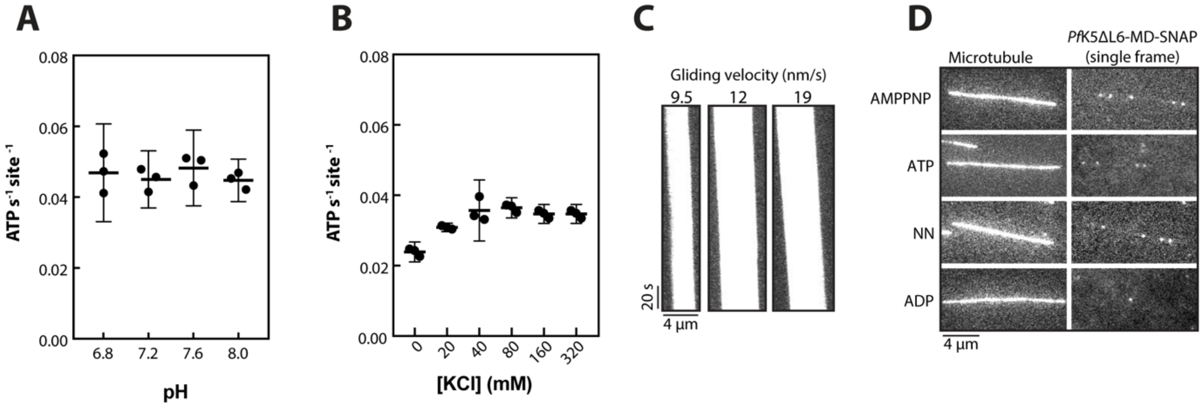

Figure S1. Biochemical behaviour of *Pf*K5ΔL6-MD constructs. (A) Effect of pH on *Pf*K5ΔL6-MD ATPase rate, with PIPES used for pH 6.8, HEPES for pH 7.2 and 7.6, and Tris-HCl for pH 8.0. n = 3 (technical replicates). The mean and 95 % confidence interval are plotted. A constant of 200 µM MTs was used. (B) As in (A), for the effect of KCl concentration on *Pf*K5ΔL6-MD ATPase activity. (C) Example kymographs from *Pf*K5ΔL6-MD-SNAP mediated MT gliding experiments, annotated with measured velocities. 0.0859 µm/pixel, and 0.57 s/pixel. (D) Raw data from *Pf*K5ΔL6-MD-SNAP single molecule MT binding experiments, with different nucleotide treatments (NN = no nucleotide). On the left, the MT reference image is shown, and on the right is an example single frame from the corresponding *Pf*K5ΔL6-MD-SNAP movie. 0.0859 µm/pixel.

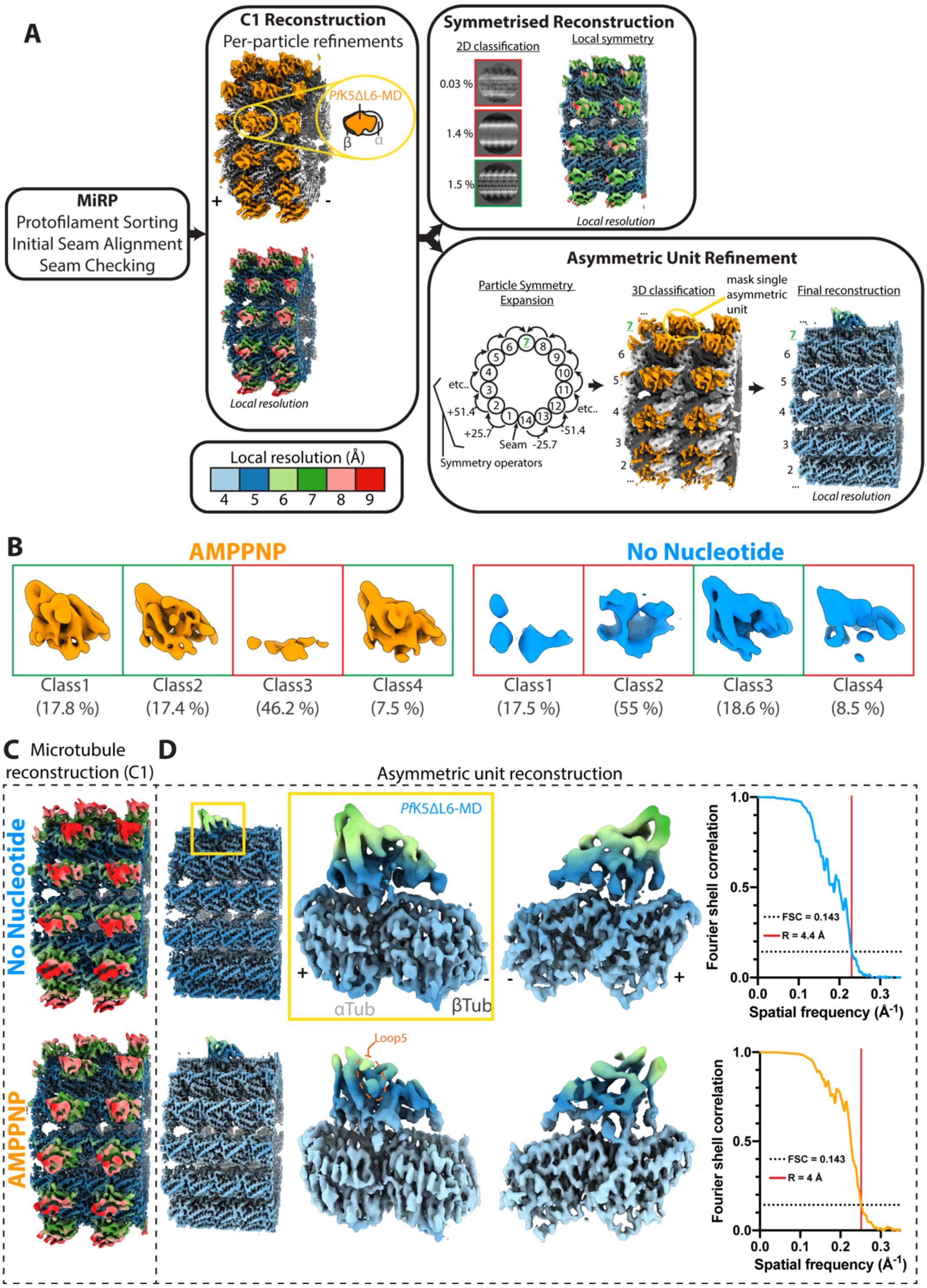

Figure S2. Cryo-EM 3D reconstruction and molecule modelling of *Pf*K5ΔL6-MD no nucleotide and AMPPNP states. (A) Overview of the 3D image processing strategy employed, with reconstructions of the *Pf*K5ΔL6-MD AMPPNP for each step shown, and coloured by local resolution. Firstly, alignment of asymmetric 14 protofilament MTs is achieved with MiRP steps. Then, a high resolution alignment and reconstruction is performed without applying symmetry, and per-particle CTF and motion correction is performed. A symmetrised reconstruction using local symmetry in RELION can then be created, and improved using 2D classification to select for optimal particles. Example 2D

classes are displayed in green or red boxes which indicate classes that were selected or discarded respectively. To improve decay in *Pf*K5ΔL6-MD resolution, an asymmetric unit refinement step is performed where symmetry expansion and 3D classification are used to obtain a more homogeneous subset of *Pf*K5ΔL6-MD motors. MT protofilament numbers are labelled 1-14, with protofilament 7 highlighted in green to indicate the location of classification mask. (B) 3D class averages from the *Pf*K5ΔL6-MD focussed classification step in asymmetric unit refinement. The red and green boxes indicate classes that were discarded or selected respectively. (C) C1 (unsymmetrised) *Pf*K5ΔL6-MD/microtubule reconstructions for the *Pf*K5ΔL6-MD no nucleotide and AMPPNP states, coloured according to the local resolution scheme in (A). (D) *Pf*K5ΔL6-MD no nucleotide and AMPPNP state structures after asymmetric unit refinement. Surface colouring is according to the local resolution scheme in (A). On the left, the central portion of the *Pf*K5ΔL6-MD/microtubule reconstructions, with *Pf*K5ΔL6-MD enriched at one site. In the middle, the *Pf*K5ΔL6-MD/αβ-tubulin asymmetric unit extracted from the microtubule reconstruction on the left. On the right, Fourier shell correlation (FSC) calculated from independently refined data halves, used to calculate overall resolution (R). FSC was calculated using a solvent mask.

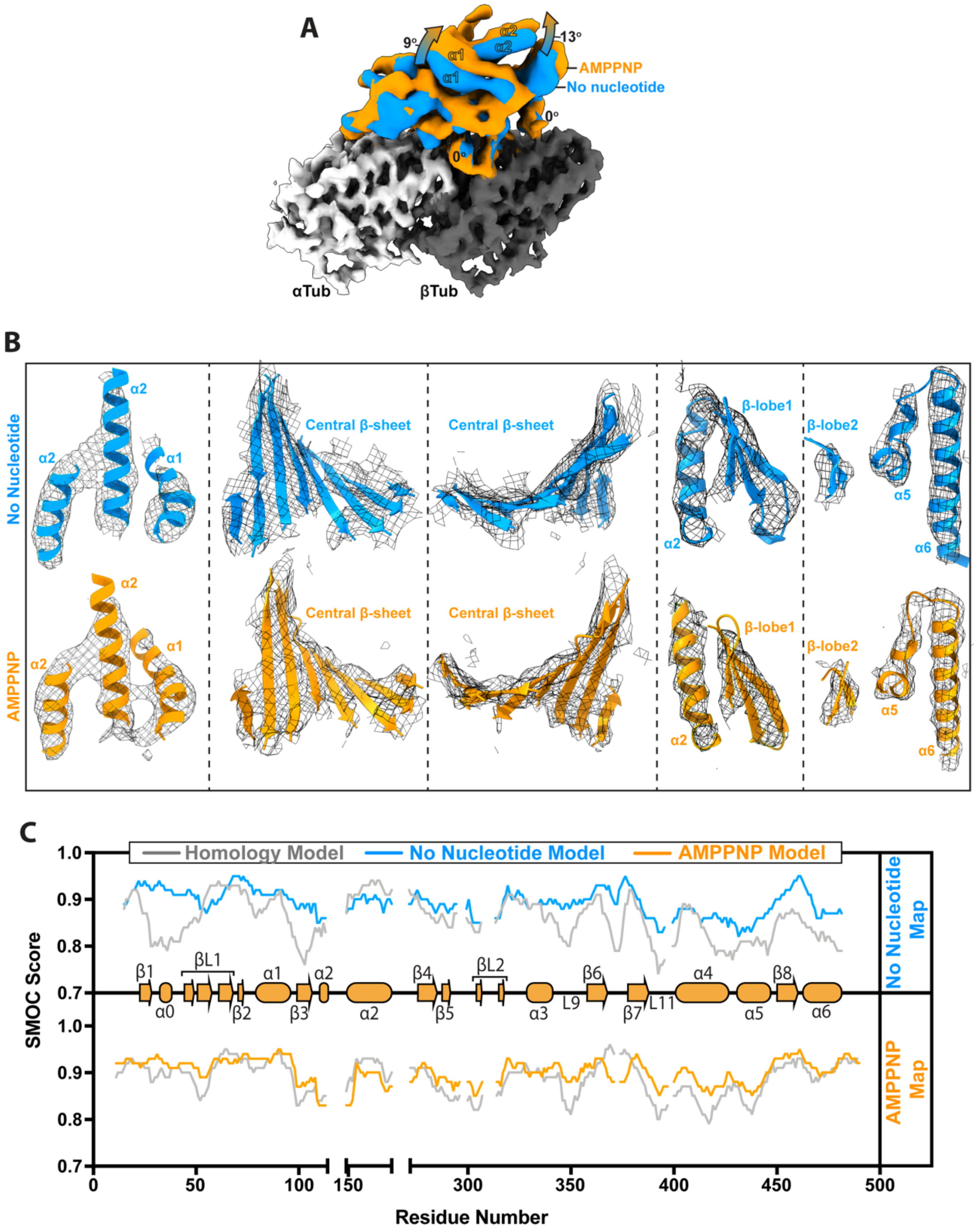

Figure S3. Secondary structure conformations of the *Pf*K5ΔL6-MD no nucleotide and AMPPNP states. (A) Alignment of the no nucleotide and AMPPNP state cryo-EM maps on αβ-tubulin, showing that conformational changes between the no nucleotide and AMPPNP state can be observed in the cryo-EM maps. (B) The fit to density of the models for various *Pf*K5ΔL6-MD secondary structure elements, showing that α-helices and β-sheets are well resolved. (C) Per-residue TEMPy SMOC scores [84,85] for the no nucleotide and AMPPNP state models, that indicate the fit of the model to cryo-EM maps. The SMOC score for the homology model is also show, to demonstrate how the flexible fitting process has improved the models fit to density.

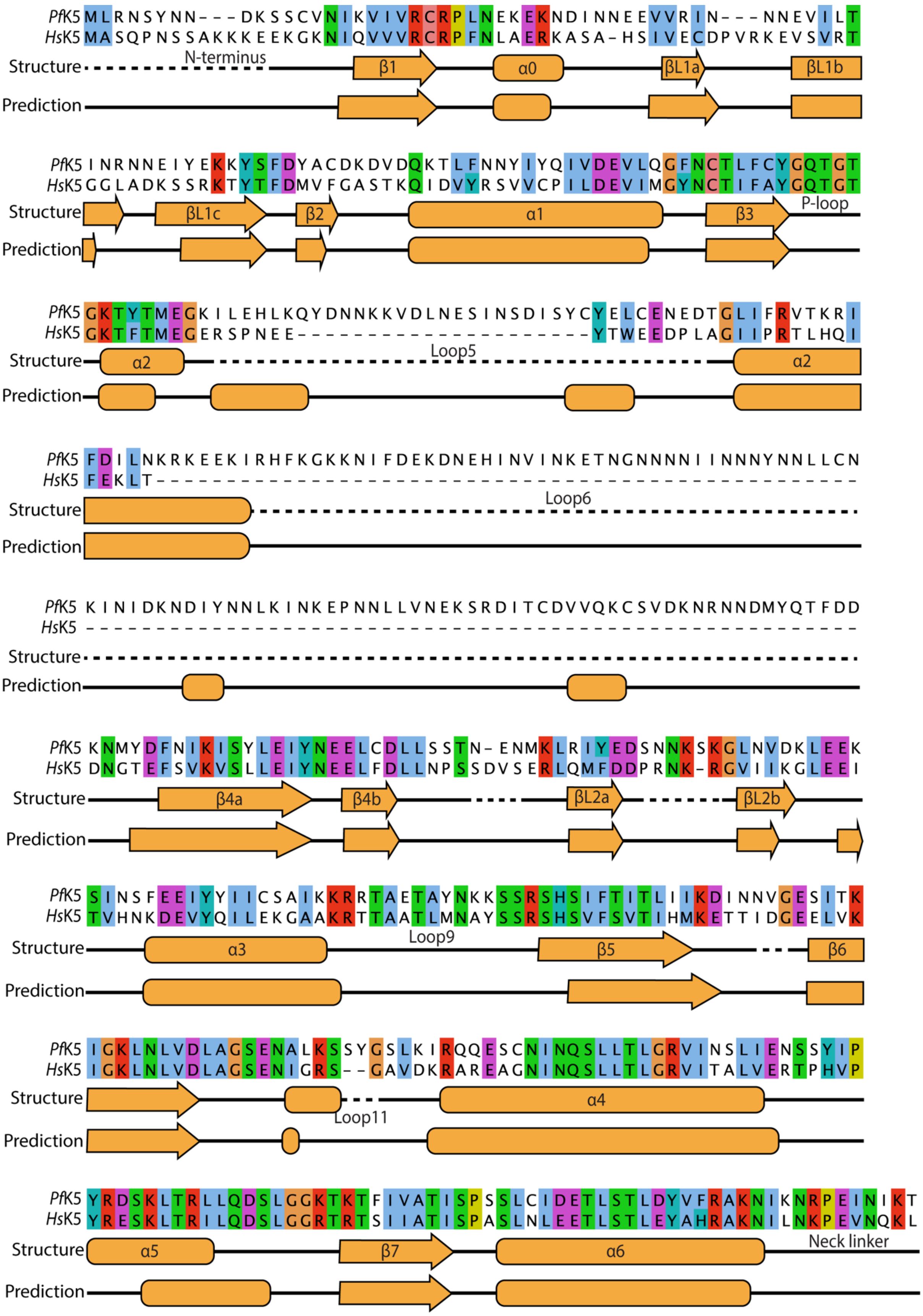
PfK5 MLRNSYNN---DKSSCVNIKVIVRCRPLNEKEKNDINNEEVVRIN---NNEVILT
HsK5 MASQPNSSAKKKEEKGKNIQVVVRCRPFNLAERKASA-HSIVECDPVRKEVSVRT
Structure
N-terminus
β1
α0
βL1a
βL1b
Prediction
PfK5 INRNNEIYEKKYSFDYACDKDVDQKTLFNNYIYQIVDEVLQGFNCTLFCYGQTGT
HsK5 GGLADKSSRKTYTFDMVFGASTKQIDVYRSVVCPILDEVIMGYNCTIFAYGQTGT
Structure
βL1c
β2
α1
β3
P-loop
Prediction
PfK5 GKTYTMEGKILEHLKQYDNNKKVDLNESINSDISYCYELCENEDTGLIFRVTKRI
HsK5 GKTFTMEGERSPNEE---------------------YTWEEDPLAGIIPRTLHQI
Structure
α2
Loop5
α2
Prediction
PfK5 FDILNKRKEEKIRHFKGKKNIFDEKDNEHINVINKETNGNNNNIINNNYNNLLCN
HsK5 FEKLT--
Structure
Loop6
Prediction
PfK5 KINIDKNDIYNNLKINKEPNNLLVNEKSRDITCDVVQKCSVDKNRNNDMYQTFDD
HsK5 ---
Structure
Prediction
PfK5 KNMYDFNIKISYLEIYNEELCDLLSSTN-ENMKLRIYEDSNNKSKGLNVDKLEEK
HsK5 DNGTEFSVKVSLLEIYNEELFDLLNPSSDVSERLQMFDDPRNK-RGVIIKGLEEI
Structure
β4a
β4b
βL2a
βL2b
Prediction
PfK5 SINSFEEIYYIICSAIKKRRTAETAYNKKSSRSHSIFTITLIIKDINNVGESITK
HsK5 TVHNKDEVYQILEKGAAKRTTAATLMNAYSSRSHSVFSVTIHMKETTIDGEELVK
Structure
α3
Loop9
β5
β6
Prediction
PfK5 IGKLNLVDLAGSENALKSSYGSLKIRQQESCNINQSLLTLGRVINSLIENSSYIP
HsK5 IGKLNLVDLAGSENIGRS--GAVDKRAREAGNINQSLLTLGRVITALVERTPHVP
Structure
Loop11
α4
Prediction
PfK5 YRDSKLTRLLQDSLGGKTKTFIVATISPSSLCIDETLSTLDYVFRAKNIKNRPEINIKT
HsK5 YRESKLTRILQDSLGGRTRTSIIATISPASLNLEETLSTLEYAHRAKNILNKPEVNQKL
Structure
α5
β7
α6
Neck linker
Prediction

Figure S4. Primary sequence alignment of *Pf*K5 and *Hs*K5. Coloured by the ClustalX scheme. Secondary structure elements corresponding to the *Pf*K5ΔL6-MD AMPPNP structure are shown, in addition to secondary structure prediction for *Pf*K5.

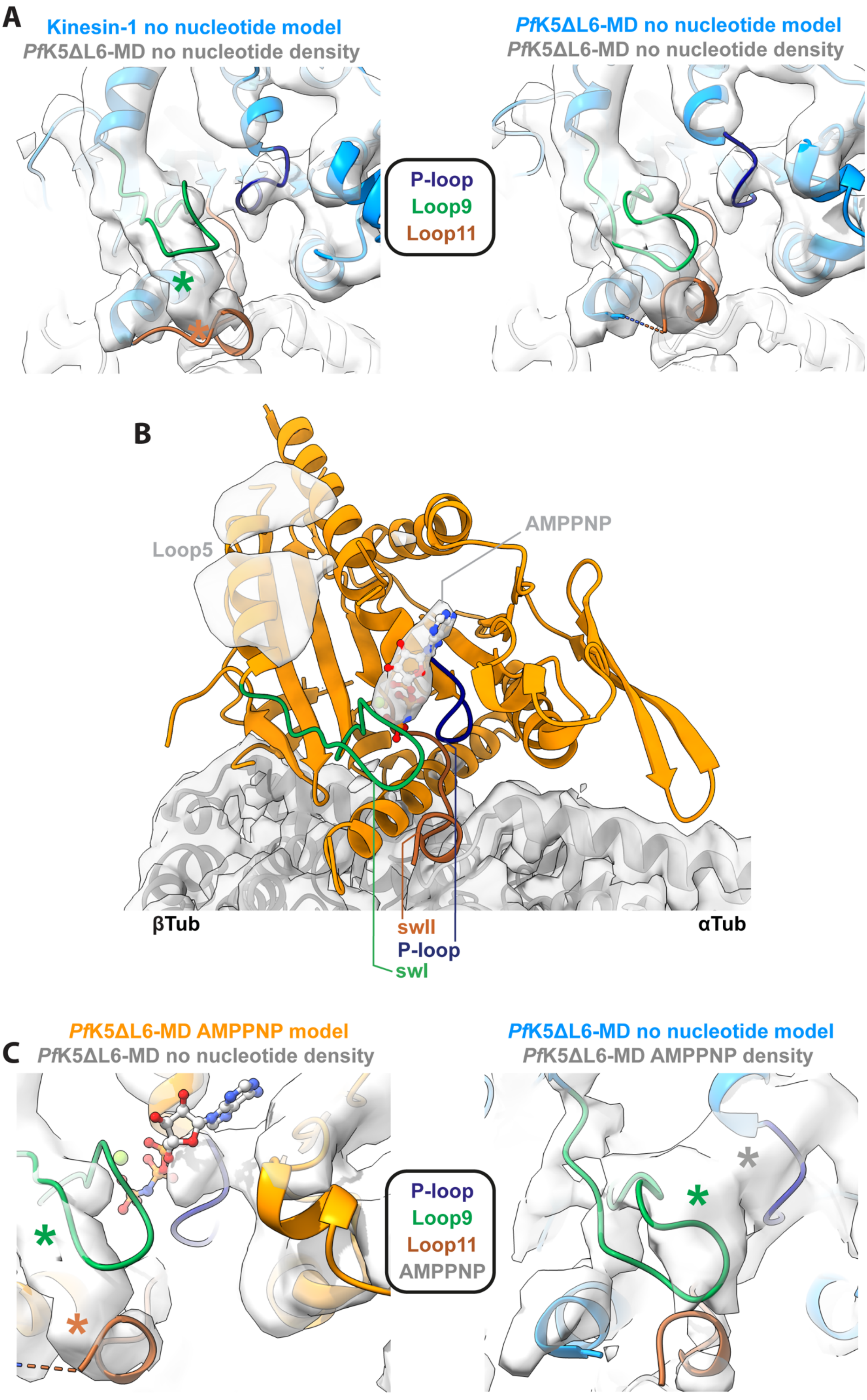

A
Kinesin-1 no nucleotide model
PfK5ΔL6-MD no nucleotide density
PfK5ΔL6-MD no nucleotide model
PfK5ΔL6-MD no nucleotide density
P-loop
Loop9
Loop11
B
AMPPNP
Loop5
βTub
αTub
swII
P-loop
swI
C
PfK5ΔL6-MD AMPPNP model
PfK5ΔL6-MD no nucleotide density
PfK5ΔL6-MD no nucleotide model
PfK5ΔL6-MD AMPPNP density
P-loop
Loop9
Loop11
AMPPNP

Figure S5. *Pf*K5ΔL6-MD nucleotide binding site. (A) To highlight differences between *Pf*K5ΔL6-MD no nucleotide switch loop structure and previous kinesin motor domain structures, the left panel shows a no nucleotide kinesin-1 cryo-EM structure (blue; PDB ID: 3J8X) rigid-body fitted into *Pf*K5ΔL6-MD no nucleotide density (grey), demonstrating that the kinesin-1 switch loops are a poor fit (indicated using coloured *). This is compared to the *Pf*K5ΔL6-MD no nucleotide model in the right panel. (B) To illustrate cryo-EM density corresponding to AMPPNP, synthetic density corresponding to the protein components of the *Pf*K5ΔL6-MD model – i.e. without AMPPNP - was calculated at 6 Å and was subtracted from *Pf*K5ΔL6-MD AMPPNP state cryo-EM reconstruction. The resulting difference density corresponds to the bound nucleotide (modelled as AMPPNP given the sample preparation conditions), αβ-tubulin, and loop5, which was not included in the model. (C) To highlight structural differences in the nucleotide binding site between the *Pf*K5ΔL6-MD no nucleotide and AMPPNP states, in the left panel the *Pf*K5ΔL6-MD AMPPNP state model (orange) is rigid body fitted to no nucleotide state density (grey), showing a poor fit to density (indicated using coloured *). In the right panel, the *Pf*K5ΔL6-MD no nucleotide state model (blue) is rigid-body fitted to *Pf*K5ΔL6-MD AMPPNP state density (grey).

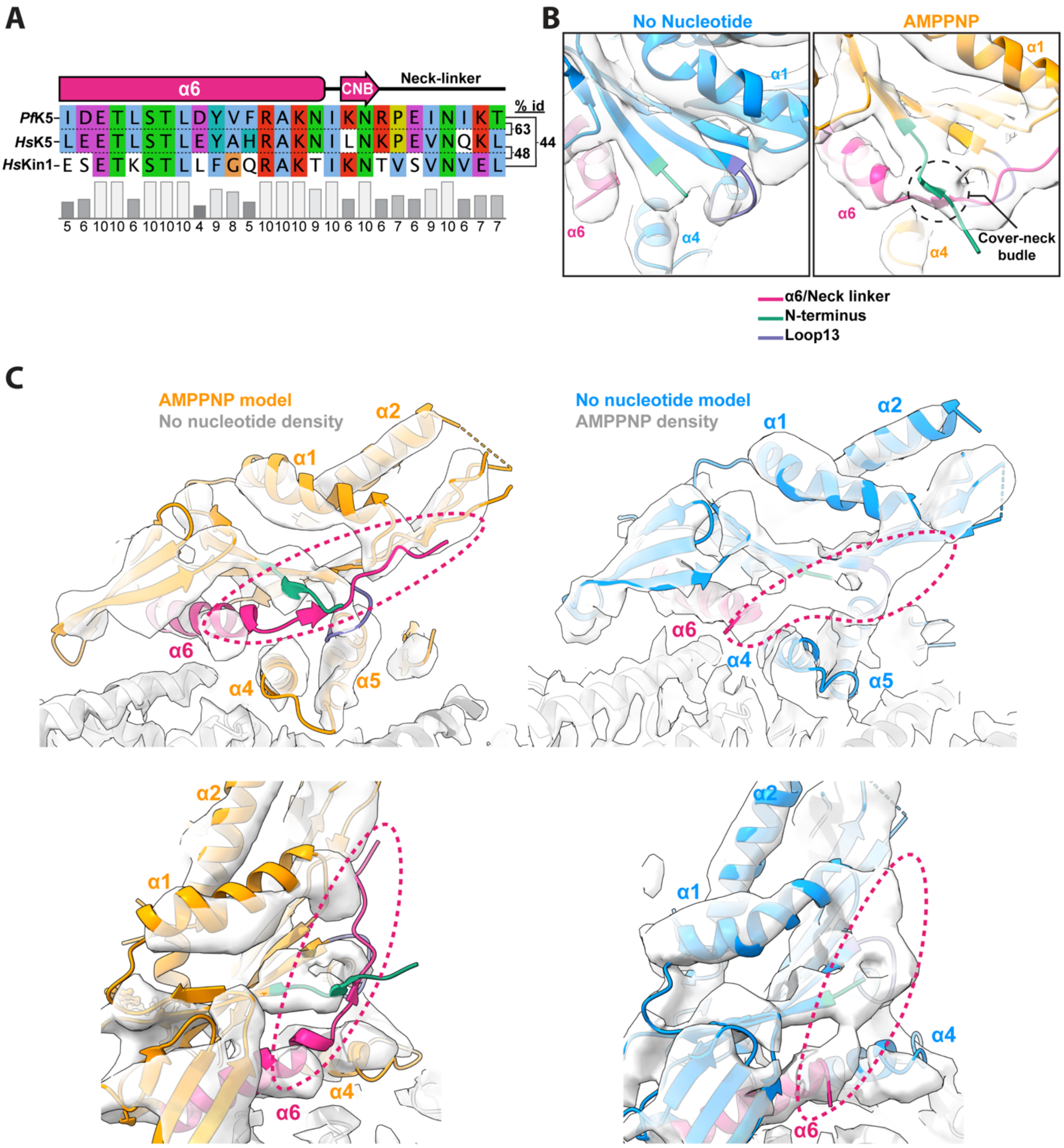

Figure S6. *Pf*K5ΔL6-MD cover-neck bundle and neck linker formation. (A) Sequence alignment of helixα6 and the neck-linker, with conservation score from Jalview below. % sequence identity between the different kinesins is noted on the right. (B) View of the *Pf*K5ΔL6-MD no nucleotide and AMPPNP reconstructions showing increased density for the N-terminus in the AMPPNP state, supporting cover neck bundle formation. (C) Demonstration of the presence of neck linker density in the *Pf*K5ΔL6-MD AMPPNP state, and its absence in the no nucleotide state. In the top and bottom left panels, the *Pf*K5ΔL6-MD AMPPNP state model (orange) is shown rigid-body fitted into *Pf*K5ΔL6-MD no nucleotide density (light grey). The *Pf*K5ΔL6-MD model was fitted into density independently of α/β-tubulin. In the top and bottom right panels the *Pf*K5ΔL6-MD no nucleotide state model (blue) is shown rigid-body fitted into *Pf*K5ΔL6-MD AMPPNP state density, with a pink dotted line demonstrating empty density for the neck linker, that is not accounted for by the no nucleotide state model.

α-tubulin

```
P. falciparum MREVISIHVGQAGIQVGNACWELFCLEHGIQPDGQMPSDKASRANDDAFNTF
   H. sapiens MRECISIHVGQAGVQIGNACWELYCLEHGIQPDGQMPSDKTIGGGDDSFNTF

              FSETGAGKHVPRCVFVDLEPTVVDEVRTGTYRQLFHPEQLISGKEDAANNFA
              FSETGAGKHVPRAVFVDLEPTVIDEVRTGTYRQLFHPEQLITGKEDAANNYA

              RGHYTIGKEVIDVCLDRIRKLADNCTGLQGFLMFSAVGGGTGSGFGCLMLER
              RGHYTIGKEIIDLVLDRIRKLADQCTGLQGFLVFHSFGGGTGSGFTSLLMER

              LSVDYGKKSKLNFCCWPSPQVSTAVVEPYNSVLSTHSLLEHTDVAIMLDNEA
              LSVDYGKKSKLEFSIYPAPQVSTAVVEPYNSILTTHTTLEHSDCAFMVDNEA

              IYDICRRNLDIERPTYTNLNRLIAQVISSLTASLRFDGALNVDVTEFQTNLV
              IYDICRRNLDIERPTYTNLNRLIGQIVSSITASLRFDGALNVDLTEFQTNLV

              PYPRIHFMLSSYAPVVSAEKAYHEQLSVSEITNSAFEPANMMAKCDPRHGKY
              PYPRIHFPLATYAPVISAEKAYHEQLSVAEITNACFEPANQMVKCDPRHGKY

              MACCLMYRGDVVPKDVNAAVATIKTKRTIQFVDWCPTGFKCGINYQPPTVVP
              MACCLLYRGDVVPKDVNAAIATIKTKRTIQFVDWCPTGFKVGINYQPPTVVP

              GGDLAKVMRAVCMISNSTAIAEVFSRMDQKFDLMYAKRAFVHWYVGEGMEEG
              GGDLAKVQRAVCMLSNTTAIAEAWARLDHKFDLMYAKRAFVHWYVGEGMEE

              EFSEAREDLAALEKDYEEVGIESNEAEGEDEGYEADY
              EFSEAREDMAALEKDYEEVGVDSVEGEGEEEGEE--Y
```

β-tubulin

```
P. falciparum MREIVHIQAGQCGNQIGAKFWEVISDEHGIDPSGTYCGDSDLQLERVDVFYN
   H. sapiens MREIVHIQAGQCGNQIGAKFWEVISDEHGIDPTGTYHGDSDLQLDRISVYYN

              EATGGRYVPRAILMDLEPGTMDSVRAGPFGQLFRPDNFVFGQTGAGNNWAKG
              EATGGKYVPRAILVDLEPGTMDSVRSGPFGQIFRPDNFVFGQSGAGNNWAKG

              HYTEGAELIDAVLDVVRKEAEGCDCLQGFQITHSLGGGTGSGMGTLLISKIR
              HYTEGAELVDSVLDVVRKEAESCDCLQGFQLTHSLGGGTGSGMGTLLISKIR

              EEYPDRIMETFSVFPSPKVSDTVVEPYNATLSVHQLVENADEVQVIDNEALY
              EEYPDRIMNTFSVVPSPKVSDTVVEPYNATLSVHQLVENTDETYCIDNEALY

              DICFRTLKLTTPTYGDLNHLVSAAMSGVTCSLRFPGQLNSDLRKLAVNLIPF
              DICFRTLKLTTPTYGDLNHLVSATMSGVTTCLRFPGQLNADLRKLAVNMVPF

              PRLHFFMIGFAPLTSRGSQQYRALTVPELTQQMFDAKNMMCASDPRHGRYLT
              PRLHFFMPGFAPLTSRGSQQYRALTVPELTQQVFDAKNMMAACDPRHGRYLT

              ACAMFRGRMSTKEVDEQMLNVQNKNSSYFVEWIPHNTKSSVCDIPPKGLKMA
              VAAVFRGRMSMKEVDEQMLNVQNKNSSYFVEWIPNNVKTAVCDIPPRGLKMA

              VTFVGNSTAIQEMFKRVSDQFTAMFRRKAFLHWYTGEGMDEMEFTEAESNMN
              VTFIGNSTAIQELFKRISEQFTAMFRRKAFLHWYTGEGMDEMEFTEAESNMN

              DLVSEYQQYQDATAEEEGEFEEEEGDVEA
              DLVSEYQQYQDATAEEEEDFGEEAEE-EA
```

βlobe-2 Helixα5 Loop12 Helixα4 Loop11 Helixα6 βlobe-1/loop2

Figure S7. Sequence conservation between *P. falciparum* and *H. sapiens* αβ-tubulin. *P. falciparum* α1-tubulin (Uniprot ID: P14642) and β-tubulin (P14643), and *H. sapiens* α-tubulin (Uniprot ID: Q71U36) and β-tubulin (P07437), were aligned in with MAFFT [66] using the L-INS-I method, and visualised in Jalview [88]. Black lines indicate the sections of tubulin that contribute to the kinesin binding site.

Kinesin motor domain secondary structure elements	Subdomain	Change In angle between no nucleotide and ATP-like states (°)
α0	P-loop	9
α1	P-loop	9
α2	switch-I/II	13
α3	switch-I/II	15
α4	MT binding	1
α5	MT binding	0
α6	P-loop	13

Table S1. Comparative rotation of *Pf*K5ΔL6-MD helices between the no nucleotide and AMPPNP states.

α-tubulin residue	β-tubulin residue	*Pf*K5ΔL6-MD (no nucleotide)	*Pf*K5ΔL6-MD (AMPPNP)	*Pf*K5ΔL6-MD secondary structure element
Glu423		Arg52	Arg52	βlobe-1/loop2
Asp424			Asn53	
Lys430			Glu55	
Glu423		Lys59	Lys59	
	Met406		Arg303	βLobe-2
	Glu407		Arg303	
	Glu410		Arg303	
	Met406		Tyr305	
	Glu410	Tyr305	Tyr305	
	Met406	Glu306		
	Glu410	Glu306		
Glu414			Ser390	Loop11
Glu414		Asn392		
Tyr108			Leu394	
	Asp161	Lys402	Lys402	Helixα4/loop12
Gly410		Gln405		
Gly410			Cys409	
Val409		Asn412	Asn412	
Gly410		Gln413	Gln413	
Val405		Leu416		
Val409		Leu416		
His406		Leu416	Leu416	
Glu415			Leu416	
Lys401		Glu427		
	Gln424		Ser430	
	Asp417	Tyr431	Tyr431	
	Ser420		Tyr431	
	Glu421		Tyr431	
	Gln424		Tyr431	
	Glu421	Ile432		Helixα5

	Asp417	Arg435	Arg435	
	Arg262	Arg435		
	Phe260		Asp436	
	Arg262	Asp436	Asp436	
	Glu194		Lys438	
	Glu410	Arg441		
	Asp417		Arg441	
Glu420			Asp467	Helixα5
Glu414		Ser471		
Ser419		Asp474		
Tyr399		Arg478		
Arg402		Arg478		
Ser419		Arg478		

Table S2. Residue-residue contacts between the *Pf*K5ΔL6-MD no nucleotide and AMPPNP states, and αβ-tubulin. Residues within contact distance were detected in Chimera.

**Parameter**	**PF Sorting**	**Initial Seam Alignment**				**Seam Check**
		**Global Search**	**Local Search**	**Rot Refine**	**X/Y Refine**	
Offset range	15	15	15	15	4	–
Offset step	1	1	1	1	0.5	
Angular sampling	1.8	0.9	0.9	0.9	0.9	
Sigma Rot	0	0	18	3	3	
Iterations	1	1	1	10	10	1
MiRP pre-run	_	Reset R, X, Y*	Reset X, Y	Reset X, Y	_	_
MiRP post-run	Vote on PF Number	Vote on Rot	Vote on Rot	Vote on X/Y shifts	–	Vote on seam position

Table S3. MiRP Alignment parameters. * R.= Rot angle, X = X-shift, Y = Y-shift.

**Start**	**End**	**Restraint**
151	172	α-helix
O1B (AMPPNP)	NZ Lys106	distance (2.7 Å)
O1G (AMPPNP)	NZ Lys106	distance (2.4 Å)
CA	NZ Lys106	distance (5.8 Å)
OE1 Glu391	NH2 Arg355	distance (2.5 Å)
OE2 Glu391	NH1 Arg355	distance (4 Å)
OD1 Asn412	N Glu391	distance (2.9 Å)
ND2 Asn412	O Glu391	distance (2.8 Å)

Table S4. Restraints used in *Pf*K5ΔL6-MD homology model generation
